# Temperature-Regulated Gating Enables Gas Separations in Ultramicroporous Aluminum Formate, ALF

**DOI:** 10.1021/acs.chemmater.5c01157

**Published:** 2025-09-02

**Authors:** Hayden A. Evans, Taner Yildirim, Gavin A. McCarver, Thuc T. Mai, Yongqiang Cheng, Zeyu Deng, Ryan A. Klein, Dan Zhao, Pieremanuele Canepa, Angela R. Hight Walker, Anthony K. Cheetham, Craig. M. Brown

**Affiliations:** † Center for Neutron Research, 10833 National Institute of Standards and Technology, Gaithersburg, Maryland 20899, United States; ‡ Physical Measurement Laboratory, National Institute of Standards and Technology, Gaithersburg, Maryland 20899, United States; § Neutron Scattering Division, 6146 Oak Ridge National Laboratory, Oak Ridge, Tennessee 37830, United States; ∥ Department of Materials Science and Engineering, 37580 National University of Singapore, Singapore 117585, Singapore; ⊥ Material, Chemical, and Computational Sciences Directorate, National Renewable Energy Laboratory, Golden, Colorado 80401, United States; # Department of Chemical and Biomolecular Engineering, National University of Singapore, Singapore 117585, Singapore; ∇ Department of Electrical & Computer Engineering, University of Houston, Houston, Texas 77204, United States; ○ Materials Research Laboratory, 8786 University of California, Santa Barbara, California 93106, United States

## Abstract

Physisorption is a reversible exothermic phenomenon where molecular kinetic energy is limited and interactions between guest molecules and materials are favored at low temperatures. However, in certain ultramicroporous materials, physisorption can be impacted by subtle structural changes on decreasing temperature that slows or even stops adsorbate diffusion, circumventing thermodynamic expectations. These unique ultramicroporous materials are described as temperature-regulated gating adsorbents and, given their special properties, can facilitate mix-and-match gas separations by simply controlling temperature. To date, though understood to be remarkably useful, there is still ambiguity about how best to identify, characterize, and rationalize the performance of these materials. To address this issue, we provide a practical analytical framework of a model gating material, Al­(HCOO)_3_ (ALF). Our work illustrates how the gating effect in ALF originates from the changing dynamics of the formate linkers that define the apertures between porous cavities. As formate dynamics increase with temperature, new kinetic adsorption regimes for an adsorbate can be accessed, marked by kinetic inflection temperatures (KITs). Identification of these temperatures allows kinetic or absolute gating separations to be devised without exhaustive experimentation. However, though an elevated temperature regime may promote fast diffusion for an adsorbate, adsorption quantities can be minimal if thermodynamics of adsorption have been overcome. By using gas sorption studies with noble gases, H_2_, N_2_, O_2_, CO_2_, and C_2_H_2_, as well as crystallography, spectroscopy, and modeling, our work elucidates how the convoluted effects of thermodynamics and kinetics affect a system like ALF and how they can be leveraged for separation design.

## Graphical abstract

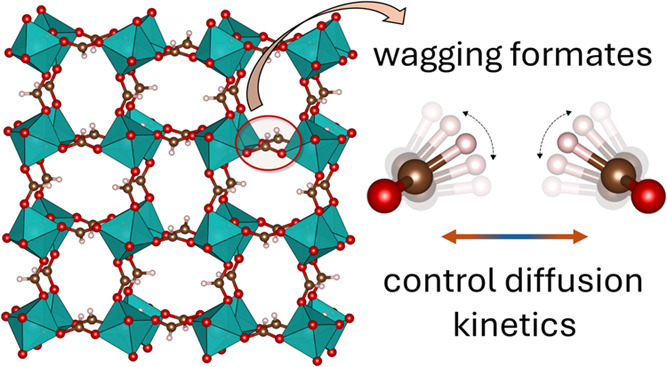

## 1 Introduction

Metal–organic frameworks (MOFs) are a large material class with impressive chemical versatility. With hundreds of possible framework types, countless organic ligands, and almost every metal in the periodic table at one’s disposal, MOFs provide a seemingly endless research field.^1–3^ Furthermore, since many MOFs are porous, they are often useful as adsorbents.^4^ All MOF adsorbents employ guest-framework interactions to drive adsorption, including van der Waals interactions, hydrogen bonding, and even binding to coordinatively unsaturated metal centers.^5^

Ultramicroporous MOFs, a subcategory of MOFs with pore widths less than 7 Å, exemplify how guest-framework interactions can be both strong and selective.^6–9^ Such materials include the SIFSIX/TIFSIX compounds,^10,11^ metal formates,^12–17^ CALF-20,^18^ NbOFFIVE,^19^ MAF-49,^20^ and numerous others with varied chemistry.^7,21–24^ Given the varied chemistry, the adsorptive applications are diverse, ranging from selective CO_2_ capture, noble gas purification, hydrocarbon separation, and more. Ultimately, the impressive properties of these materials are coupled to their confined channel structures, which create substantial and selective interactions between adsorbates and the MOF framework. However, given that the adsorbates are often commensurate in size with pore diameters within the crystal structures, ultramicroporous MOFs (and specific zeolites) have also been known to display anomalous physisorptive behavior when compared to MOFs with large pores.^15,25–28^ This is often temperature dependent, where adsorption properties, especially adsorption kinetics, can evolve unexpectedly as diffusion becomes inhibited.

An example of seemingly anomalous behavior in ultramicroporous materials includes when physisorption maxima occur at warmer temperatures and physisorption minima occur at cooler temperatures, despite this being an exothermic process. This inverted behavior has been documented in ultramicroporous MOFs, such as Mn­(HCOO)_2_ and Cu­(OPTz), and was attributed to the changing diffusion pathways as the positions of organic linkers adjusted with temperature.^15,29^ The behavior was also specifically covered by May and co-workers with trapdoor zeolites, select supramolecular host calixarenes, and MOFs.^30^ Such materials are now known as temperature-regulated gating adsorbents, though the list of materials currently meeting the classification is short, as discussed in the recent review by Webley and co-workers.^31^ In terms of functionality, it was shown for trapdoor zeolites that the alkali cations allow adsorbate diffusion at specific temperatures when specific cation-adsorbate interactions open pathways through the crystal structure. However, in other zeolites,^32^ as well as for MOFs, it is now appreciated that the temperature-regulated gating phenomenon is more generally determined by framework dynamics and flexibility.^31,33–35^ Specifically, as ligands rotate/vibrate with increasing temperature, pathways through a material structure change.^36–38^ Of note, recent works from Gu, Kitagawa, and co-workers illustrate how ultramicroporous MOFs can leverage subtle structural dynamics for separating compounds with nearly identical physical properties. This includes H_2_O from D_2_O,^39^ CO_2_ from C_2_H_2_,^40^ and C_3_H_6_ from C_3_H_8_.^41^ In each work, diffusion of one of the adsorbates in the pairs is more hindered than the other, allowing effective kinetic separations to be performed.

As these remarkable gating materials can facilitate multiple gas separations via temperature tuning, understanding their varied performance and how to best characterize them to establish design criteria, is of great significance. To expand the current characterization methodology and design criteria for such important materials, we have constructed a detailed model for the high-performing temperature-regulated gating material aluminum formate, Al­(HCOO)_3_ (a.k.a. ALF, Figure 1).^42–46^ In this work, we illustrate how targeted experimental and computational studies of noble gases, H_2_, N_2_, O_2_, CO_2_, and C_2_H_2_ in ALF illuminate the temperature-regulated gating response, and how the origin of such behavior is rooted in the thermally activated dynamics of formate anions that gate channel apertures. Furthermore, we reveal how these dynamic formates manifest kinetic inflection temperatures (KITs) with adsorbates, delineating the kinetically accessible sorption regions that can be leveraged for efficient separation design. These KITs can be experimentally resolved via sorption studies but can also be predicted from nudged elastic band (NEB) density functional theory (DFT) calculations when calibrated via temperature desorption measurements of noble gases. Our developed KIT analysis shows for the first time how to isolate and rationalize the important tipping point temperatures for ultramicroporous materials, where the kinetics of diffusion and the thermodynamics of adsorption trade places in importance as the temperature increases.

**Figure 1:**
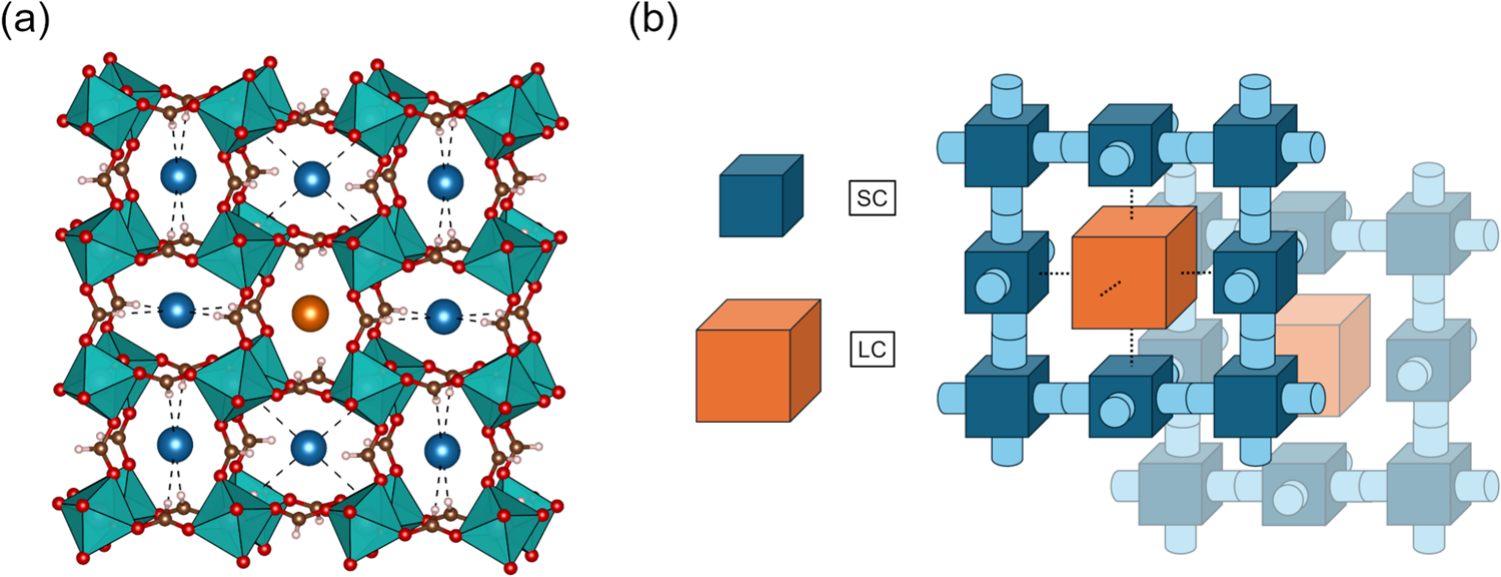
(a) Average crystal structure of ALF as resolved from neutron powder diffraction (NIST, BT-1) Rietveld analysis at 300 K.^42^ The two cavities within ALF, denoted as the Small Cavity (SC) and Large Cavity (LC) are distinguished by different color spheres (idealized adsorbates, blue for SC, orange for LC) within their cavities. The SCs, given their distinctive four inward pointing formate hydrogens, have been drawn with dashed lines toward their ideal adsorbates to emphasize how these interactions can exist, and how SC orientation changes through the crystal structure. As a function of the crystal symmetry (Im3̅), the ratio of SCs to LCs is 3:1. (b) Cartoon illustration of how ALF’s SCs and LCs arrange throughout the crystal structure. In the cartoon, colored cubes represent each cavity (dark blue = SC, orange = LC). To traverse from one cavity to the next, an adsorbate must move through an aperture defined by formate hydrogens (a gate). These are depicted as the light blue colored cylinders and the dotted lines, denoting the SC-SC gates and SC-LC gates, respectively. As can be seen, each SC has four adjacent SC’s and two LCs, and each LC has six adjacent SCs.

## 2 Results and Discussion

### 2.1 Crystal Structure of ALF

ALF is a porous material with an ReO_3_-type crystal structure (Figure 1a).^47^ It has two crystallographically distinct cavities, denoted as the Small Cavity (SC) and the Large Cavity (LC). The cavities are distinguished by how the formate hydrogens orient toward the center of each cavity: inward for the SC or outward for the LC (Figure 1a). Furthermore, as shown in Figure 1a—the placement of the formate hydrogens is significant, as they physically define the apertures between neighboring cavities and how each cavity interacts with adsorbates. From previous crystallographic examination of ALF, there is no evidence for interconversion between the two cavities as a function of temperature, which would be clearly seen from a change in its body-centered symmetry.^42^ Figure 1b illustrates how these cavities and gated apertures are arranged throughout the crystal structure. There are three times as many SCs than LCs in ALF (space group Im3̅), and between every cavity, there exists a gated aperture (depicted as cylinders or dashed lines). Depending on if an adsorbate is traversing from an SC to another SC, or an SC to an LC, a different gated aperture (and subsequent penalty) will be encountered. These distinct cavities and pathways between them prove paramount to ALF’s temperature-regulated adsorption behavior, which is verified through sorption studies.

### 2.2 Adsorption and Temperature-Programmed Desorption Studies with ALF

A clear indication of temperature-regulated gating behavior is the observation of abnormal gas isotherms, namely when adsorption isotherms exhibit an effective physisorption maximum within a specific temperature range. Below that range and above the condensation temperature of the tested adsorbate, adsorption diminishes, contrary to what would be expected thermodynamically. This effect can be appreciated from select isotherms and adsorption maxima of H_2_, O_2_, and CO_2_ with ALF, as shown in Figure 2a–f. For each gas, there is a “peak” adsorption temperature range, as well as a point where, if the temperature is too low, adsorption becomes kinetically limited or stops altogether. Generally, pronounced hysteresis between adsorption and desorption traces can indicate kinetic limitations.

**Figure 2:**
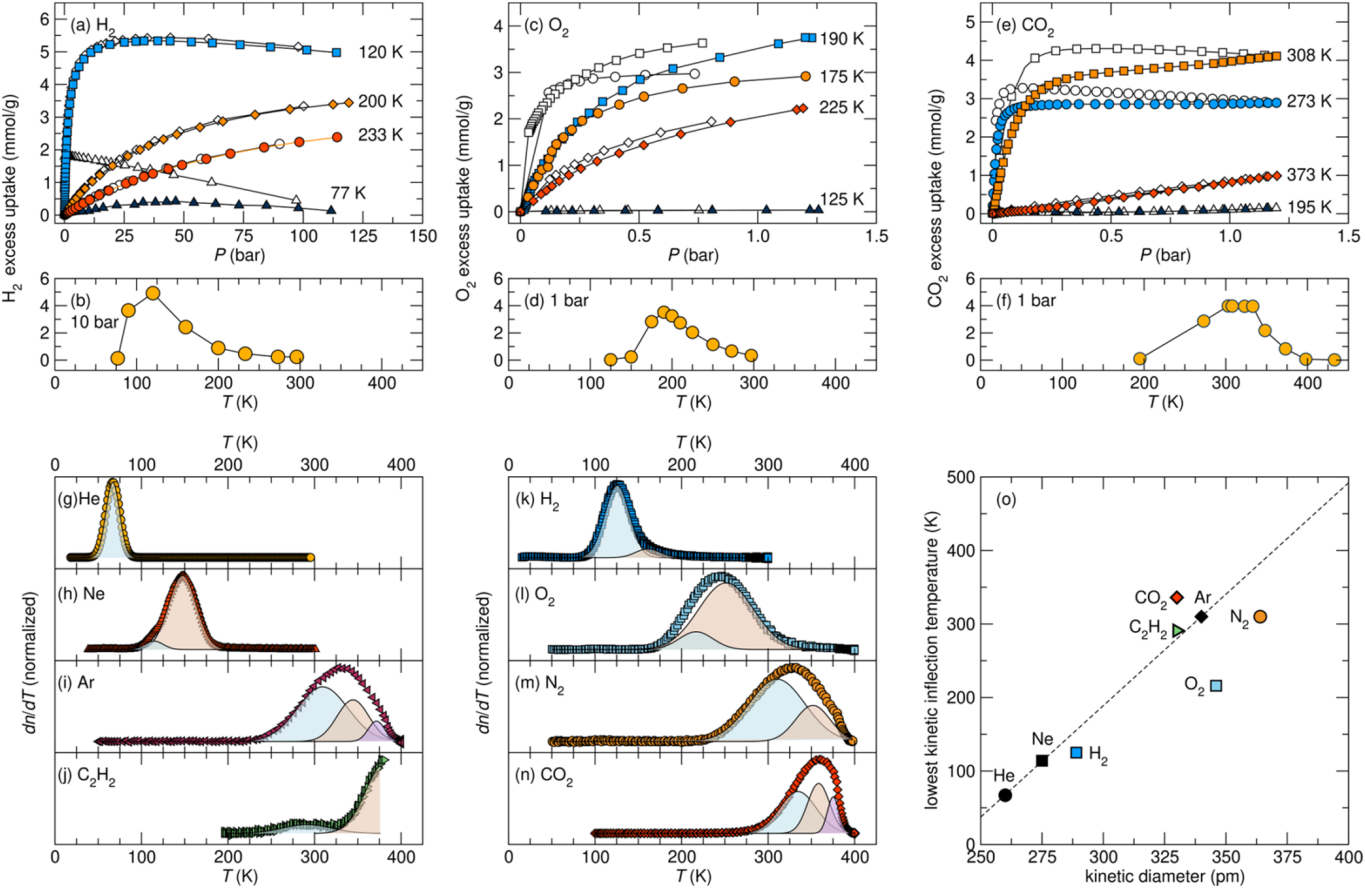
Sorption studies of ALF. (a–f) Select adsorption/desorption isotherms and effective adsorption maxima for H_2_, O_2_, and CO_2_ illustrating the hallmark adsorption properties of a thermally regulated gating material, where if temperatures are too low, kinetic limitations can prevent appreciable gas uptake on practical experimental time scales (≈ hours). The closed symbols in panels (a, c, and e) denote adsorption traces, and the open symbols denote desorption traces for a given temperature. Panels (b, d, and f) specifically illustrate effective adsorption maximum ranges for each gas with ALF at a constant pressure. 1 bar = 100 kPa. (g–n) Derivatives and composite peak fitting of normalized amount of gas released (*n*) in temperature-programmed desorption (TPD) measurements for He, Ne, Ar, C_2_H_2_, H_2_, O_2_, N_2_, CO_2_. Data normalized to each TPD maximum. A kinetic inflection temperature (KIT) is designated as the center of one of the composite peaks. (o) Graph of lowest temperature KITs vs kinetic diameter for all gases tested with ALF. The lowest KITs for all gases are He ≈ 67 K, Ne ≈ 114 K, Ar ≈ 310 K, C_2_H_2_ ≈ 291 K, H_2_ ≈ 123 K, O_2_ ≈ 216 K, N_2_ ≈ 310 K, and CO_2_ ≈ 336 K. Pertinent measurements such as other adsorption/desorption isotherms, and values of each resolved KIT, can be found in the Supporting Information (Figures S4 through S10, Table S2). The uncertainty of each data point is smaller than the symbols used.

From the isotherms of H_2_, O_2_, and CO_2_ with ALF, we can infer that ALF likely has a favorable temperature range of adsorption for other sorbates. However, effectively establishing or predicting these temperature ranges for numerous gases is time-consuming from isotherm measurements alone, as data can take hours (or even days) to collect at temperatures below the peak adsorption range. From our work on ALF, we found temperature-programmed desorption (TPD) measurements provide an efficient methodology to establish these optimal temperature ranges.^48^ Furthermore, we also found that TPD measurements not only expose these ranges, but simultaneously reveal the specific temperatures within these ranges that mark when kinetic barriers caused by ALF’s gating mechanism are overcome.

TPD measurements are routinely used to establish how and at what temperature an adsorbate will desorb from a material under vacuum. The general procedure is straightforward: first, the adsorbent is exposed to the desired adsorbate at an elevated temperature and the system is then cooled to a temperature near the adsorbate’s liquefaction/solidification point; second, the system is evacuated and further cooled to the base temperature of the apparatus; finally, the system pressure is monitored upon heating, specifically for pressure increases, marking desorption events. For many large pore adsorbents, the temperature of peak desorption can approximate the temperature at which the heat of adsorption of an adsorbate onto that surface is overcome by entropic effects (Boltzmann function dependent).^49^ For ALF, however, we found that the desorption temperatures seen in TPD measurements aligned with where pronounced hysteresis emerged between adsorption and desorption isotherms for all adsorbates.

In all TPD measurements of adsorbates with ALF (except He), we found that the derivatives of the TPD measurements were best fit using multiple Gaussian functions, as can be seen for the noble gases in Figures 2g–i (fitting denoted by colored Gaussian peaks). As we clarify later, each Gaussian within a TPD derivative represents the temperature at which a gating migration barrier within ALF is overcome for a sorbate. We refer to the center of each Gaussian peak as the Kinetic Inflection Temperature, KIT. While each KIT corresponds to a specific kinetic regime for a given gas, adsorption can still occur below the lowest KIT value, albeit with hindered diffusion. However, at a certain point below the lowest KIT, specific to each adsorbate, absolute gating occurs, wherein little to no adsorption takes place on a reasonable time scale. We discuss later how kinetic and absolute gating can be leveraged for separations.

As the gating effect is conceptually like a ball passing through a partially opened aperture, we hypothesized that the noble gases, with their isotropic atomic nature and similar chemistries, would show predictable KIT behaviors with temperature. This proves to be the case, and the general trend for He, Ne, and Ar with ALF is that as the kinetic diameter of a noble gas increases, so too does its lowest temperature KIT. This can be seen with the trend line drawn in Figure 2o, which relates each noble gas’s lowest temperature KIT to its kinetic diameter (goodness-of-fit value, *R* ^2^ = 0.9992). However, though this trend is coupled to thermodynamics, where the interaction energy between the gas and the sorbate increases in the sequence, He < Ne < Ar, gating effects prove more significant for KIT temperature. This can be appreciated by the fact that even when adsorption of Ne and Ar is thermodynamically favored at low temperatures, each gas is gated completely when temperatures reach a critical point, akin to what is seen for O_2_ and CO_2_ in Figure 2c–f. Ultimately, the TPD data of He, Ne, and Ar illustrates the simplest impact of the gating trends with ALF and how the size of the aperture into the ALF cavities is likely widening with increasing temperature, facilitating diffusion of increasingly larger “spheres”.

For adsorbates that are not isotropic spheres, such as the diatomic molecules H_2_, O_2_, and N_2_, the triatomic CO_2_, or the tetratomic C_2_H_2_, the KIT trend is more nuanced. Figure 2j–n shows the TPD derivatives for C_2_H_2_, H_2_, O_2_, N_2_, and CO_2_. In Figure 2o, if we compare the lowest KIT of every adsorbate tested versus their respective kinetic diameters, we observe that the diatomic molecules fall off the noble gas trend line and display a weaker linear correlation among themselves (not graphed, but *R* ^2^ = 0.9121). Furthermore, the adsorbate CO_2_ remains an outlier of the trends set by both the inert gases and the diatomic molecules, with its lowest temperature KIT being much *higher* than its kinetic diameter would lead us to expect. The tetratomic C_2_H_2_ KIT is just above the noble gas trend, though it would be difficult to predict that *a priori*.

To understand the varied trends of the diatomic, triatomic, and tetratomic molecules and how KITs can be leveraged for separation design of such molecules in gating materials, we will now delve into ALF’s dynamic crystal structure. We use crystallography, spectroscopy, and computation to understand ALF’s dynamics, and how the physical gates defined by ALF’s formate ligands are responsible for modulating the diffusion of gas molecules through the apertures between neighboring cavities.

### 2.3 Crystallographic and Spectroscopic Studies of ALF

From sequential Rietveld refinements with neutron and X-ray powder diffraction data between 14 and 377 K, we find that the structural behavior of ALF as a function of temperature is typical of most solid materials, including other ReO_3_-type materials.^47,50^ Namely, as the temperature increases, ALF’s lattice parameter increases after a brief period of near zero/negative thermal expansion at temperatures in the range 10 to 50 K (Figure S18). The Debye–Waller values of all elements (Al, H, C, O) behave similarly (Figure S18). However, we observe no first-order phase transition or dramatic structural change over this temperature range, as seen with some gating materials, like ZIF-7/ZIF-8 or the MIL-53 compounds.^51–56^ As diffraction gives a time averaged view of crystal structures, and given we see no major structural events that would explain its adsorption behavior, this suggests that subtle changes on the local scale likely drive the temperature-regulated gating properties of ALF.

To probe these subtle changes, we utilized variable temperature Raman and neutron spectroscopy. Figure 3 displays raw data and fitting results from temperature-dependent Raman spectra of ALF. Figure 3a shows the raw spectra of ALF between 10 and 293 K, while Figures 3b,c show the energetic position and full width at half-maximum (fwhm) change of select modes (labeled **1–7**) between 10 and 150 K. As shown in Figure 3b, modes **1–7** show minor frequency changes up to 50 K, above which slight red-shifting begins. This red-shifting is expected, given the positive thermal expansion of ALF above this temperature, as noted from diffraction studies (Figures S18, S22, and S23).^19^ However, the modes that undergo the largest frequency changes are those pertaining to the wagging and twisting of the formate anions (modes **1–4**), starting between 50 and 75 K. In Figure 3c, the unique significance of the wagging formate anions becomes apparent, as although the fwhm of every other peak follows a similar percent change trajectory (between 20 and 40% from 10 to 150 K), the fwhm of the wagging modes, specifically mode **3**, changes by ≈140%. For context, increasing fwhm of a peak in Raman spectra is related to increasing molecular fluctuation, depending on the mode.^57,58^ This same trend was also seen for ALF via inelastic neutron scattering (INS, Figure S26) and quasielastic neutron scattering (QENS, Figure S27).

**Figure 3:**
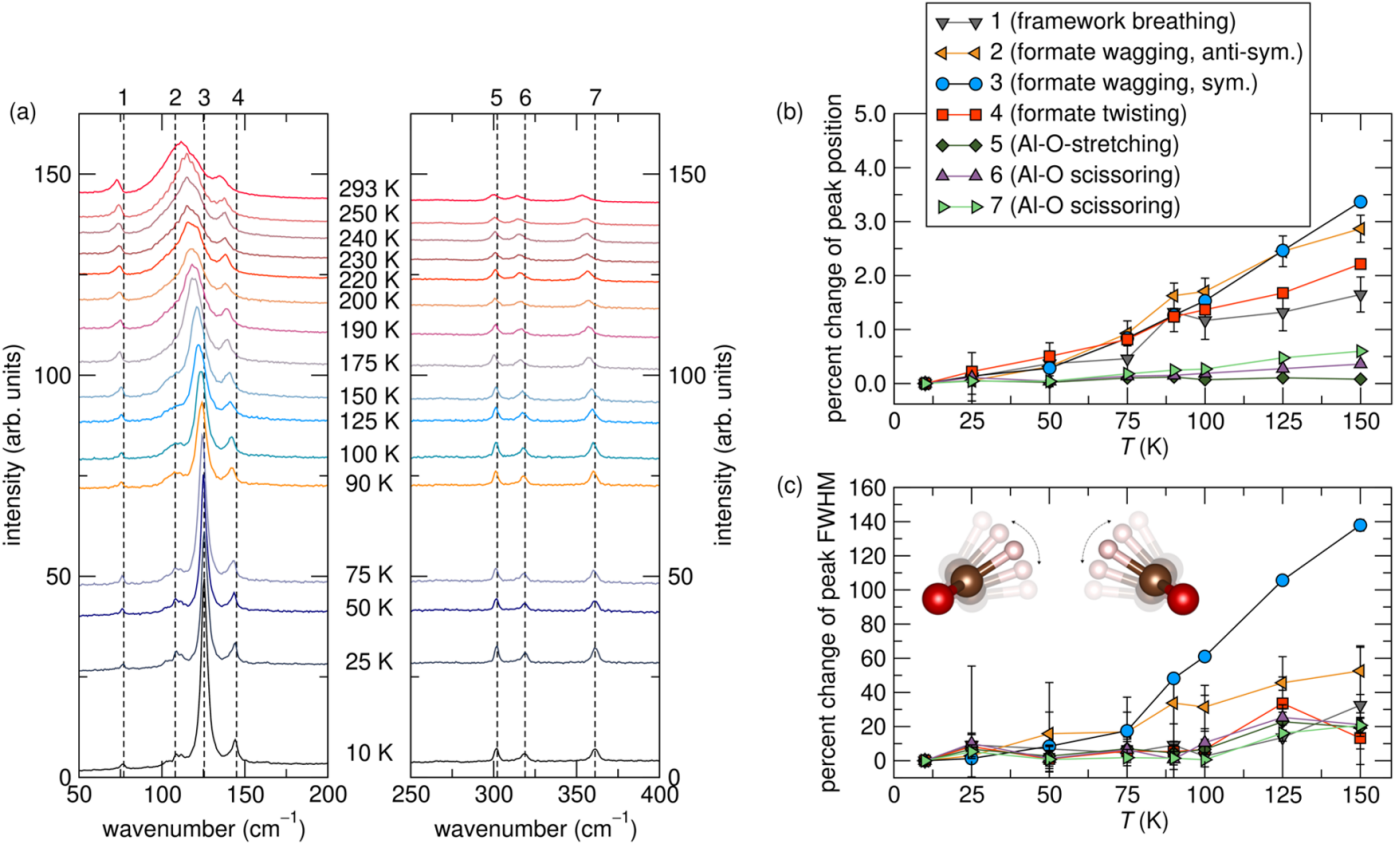
Raw data and results from fitting temperature-dependent Raman spectra of ALF. (a) The Raman spectra of ALF, highlighting areas of interest: 50 to 200 cm^–1^ (left) and 250 to 400 cm^–1^ (right). Laser λ = 515 nm. Modes of interest are labeled as **1–7**, with lines showing the approximate position of where each mode is found at 10 K included to guide the eye. Mode assignment made via DFT calculations (see Supporting Information, Table S5). (b, c) Peak fitting results of 10 to 150 K Raman data, presented as a function of percent change in (b) peak position and (c) fwhm. The sign of the percent change of the fwhm indicates the nature of change; a positive value denotes an increasing fwhm (broadening), whereas a negative value denotes a decreasing fwhm (sharpening). The inset figure illustrates how two wagging formates can change a gating aperture. Peak fitting was completed between 10 to 150 K, as peaks above 150 K begin to overlap more significantly (specifically the formate wagging cluster, **2**) to where they cannot be confidently placed. Each peak fit, difference, and peak contribution plots can be found in the Supporting Information (Figures S24 and S25). Error bars signify 1 sigma, and where not seen, are smaller than the symbols used.

If we now combine our diffraction-based understanding of the “average/static” crystal structure of ALF with its true dynamics, we can confirm the origin of ALF’s temperature-regulated gating behavior. Specifically, because the framework dynamics of ALF increase with temperature, particularly the wagging of the formate ligands, the physical apertures between cavities are likely also expanding (on average) with increasing temperature. These expanding apertures can affect the diffusion of each adsorbate differently depending on the adsorbate’s size, geometry (beyond the spherical kinetic diameter approximation), and chemical nature. Using the TPD measurements as a proxy, but verified through isotherms, we can see that for the noble gases, even the smallest of them, He, is too large to pass quickly through the aperture until the onset of the formate wagging begins near 50 K (see Figures 2g and 3b,c). As the temperature increases and the wagging of the formates become greater, the aperture becomes large enough for Ne diffusion to begin at ≈75 K (Figure 2h) and Ar diffusion at ≈230 K (Figure 2i).

The same general trend is seen with the diatomic molecules (H_2_, O_2_, and N_2_), where we can estimate from TPD results that the hindered diffusion through ALF begins at ≈90 K, ≈160 K, and ≈220 K, respectively (Figures 2k–m). For C_2_H_2_, hindered diffusion can begin near 225 K. However, the fact that the onset of diffusion with CO_2_ is at an even higher temperature (≈275 K) than expected given its kinetic diameter (Figure 2o), suggests that CO_2_ behaves differently than the noble gases and the diatomics with ALF. There are multiple potential reasons for such behavior, including electrostatic interactions, but we will provide evidence that CO_2_ fundamentally changes the vibrations of the formate linkers, altering gating behavior.

### 2.4 Density Functional Theory Calculations of Binding Energies and Kinetic Barriers

Although the interactions between adsorbates and the ALF framework are complex combinations of thermodynamic and kinetic factors, they can be accurately captured and rationalized through DFT calculations. In Figure 4, we show the analysis surrounding adsorbate-framework interactions in ALF by examining thermodynamic adsorption energies and kinetic barriers of gas migration via periodic DFT calculations. Details of the methodology are given in the Supporting Information. In Figure 4a, we highlight thermodynamic effects by illustrating how the calculated adsorption energies follow a trend based on molecular size. As the molecular size increases, so too does adsorption energy. This trend holds for both the noble gases He < Ne < Ar, the diatomics H_2_ < O_2_ < N_2_, the tetratomic C_2_H_2_, and the triatomic CO_2_, which has the largest adsorption energy of all. The orientational dependence of adsorption energy is also important for the polyatomic molecules, as exemplified by CO_2_, where favorable hydrogen bonding interactions in the small cavity SC_*z*_ orientation promote strong adsorption.^42^

**Figure 4:**
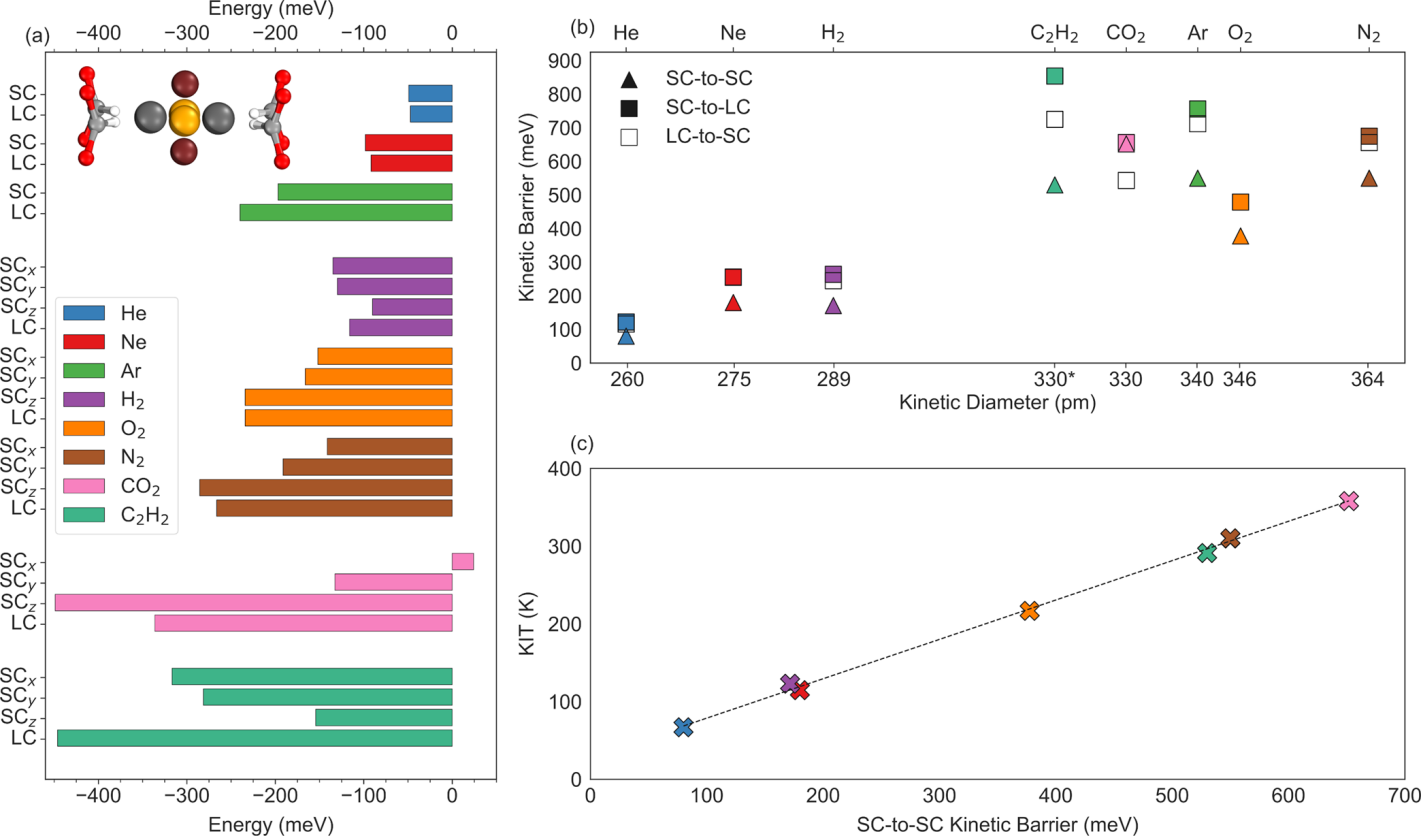
Density Functional Theory analysis of the thermodynamic adsorption energies and kinetic barriers for gas migration in ALF. (a) Binding energies for the adsorbate gases in ALF. The inset shows the orientations with respect to a small cavity (SC) described as SC*_X_* (gold spheres), SC*_Y_* (brown spheres), and SC*_Z_* (gray spheres) in the SC of ALF. (b) Kinetic barriers of gas migration from SC-to-SC (triangles), SC-to-LC (filled squares), and LC-to-SC (empty squares). C_2_H_2_ has the same kinetic diameter as CO_2_ and has been offset for clarity. (c) Kinetic barriers of gas migration from SC-to-SC are plotted against the lowest temperature KIT for each adsorbate except CO_2_, given its SC-to-SC barrier is higher. The trend line is a linear fit to all data (*R* ^2^ = 0.9977). In panel c, the value for Ar is identical to N_2_, and cannot be seen. The adsorption energies and kinetic barriers presented here can be found in kJ/mol in Tables S5 and S6 in the Supporting Information.

Recognizing the specific cavity arrangement within ALF (Figure 1), and how the formate hydrogens are dynamic and gate pathways between the SCs and LCs, we calculated kinetic barriers of gas migration using the nudged elastic band (NEB) method to probe how gas molecules traverse the distinct apertures.^59–61^ What our NEB calculations reveal is that the chemical interactions between the adsorbates and the ALF framework strongly influence the kinetic barriers of gas migration for adsorbates through these gates (Figure 4b), but also closely replicate the experimentally observed KIT trend shown in Figure 2o. In Figure 4b we show that the lowest calculated barrier for all but one adsorbate occurs when traversing the SC-to-SC gate, suggesting that this is the predominant rate-limiting diffusion pathway. The exception to this trend is for CO_2_, where the LC-to-SC barrier has the lowest calculated barrier due to the large differences in adsorption energies between the two cavities. Furthermore, rotation must also occur prior to translation to minimize energetic penalties for CO_2_ and other polyatomic molecules (Figures S28–S30).

Plotting the minimum KITs versus the SC-to-SC kinetic migration barriers for all the molecules (Figure 4c) reveals a near-perfect linear relationship (*R* ^2^ = 0.9977) that connects our computational and experimental findings. This strong correlation highlights the predictive power of a simple computational model and the underlying physical phenomena driving ALF’s observed temperature-regulated gating behavior. Specifically, the emergence of KITs is fundamentally governed by migration barriers between cavities, and in ALF, the SC-to-SC barrier appears to be the most consequential. As was noted earlier, the TPD measurements of all gases (except He) required multipeak derivatives to be fit (Figure 2). This is caused by the inhomogeneous gating structure of ALF, specifically the different barriers between the SC-LC gates and the SC-SC gates (Figures 4b and 5a,b). As shown in Figure 4b, there is a SC-to-SC barrier, and two LC-SC barriers given travel is not symmetric between the LC and SC, though the LC-SC/SC-LC energy barriers frequently overlap (Figure 4b). This is also reflected in TPD derivative fits, where two Gaussian peaks are usually sufficient for fitting the derivatives instead of three. The exceptions to this are for Ar and CO_2_, which is expected for Ar, given the calculated energy spread, but not for CO_2_. We discuss the origins of this deviation later. Lastly, we note that this strong connection between experiment and computation has also been verified through more detailed kinetic gas adsorption studies and confirms how TPD-identified KITs, experimentally obtained activation energies of diffusion, and calculated kinetic migration barriers are all linearly related (Figure S1). The power of the TPD measurements for identifying KITs though is that they can be done quickly for a small subset of gases to estimate performance of other gases, instead of kinetic gas adsorption studies, which are far more complicated and time-consuming to perform.

**Figure 5:**
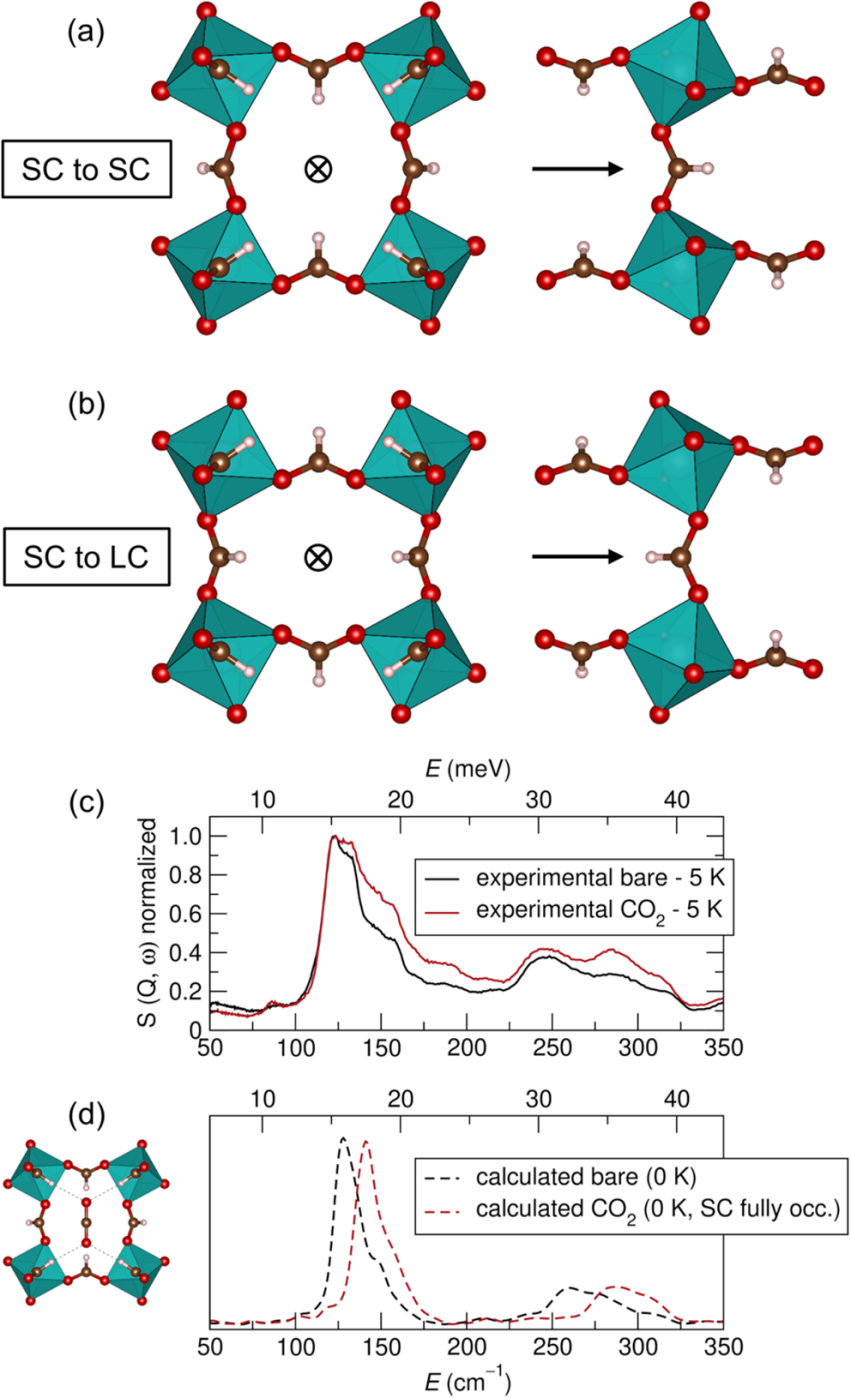
(a) Structural view of the SC-SC gate from the front, and the side. The arrow and circle with a cross (signifying the direction of the arrow into the page), denote the direction of view. (b) Structural view of the SC-LC gate from the front, and then the side. The arrow and circle with a cross (signifying the direction of the arrow into the page) denote the direction of view. The arrow in (b) shows the gate when traversing from the SC to the LC. (c) *In situ* inelastic neutron scattering (ORNL, VISION) with activated ALF, and with adsorbed CO_2_. Intensity is normalized to the spectra maximum. (d) DFT Simulated INS spectra for ALF with and without CO_2_. Intensity is normalized to the spectra maximum. The simulated CO_2_ spectra is for when the SC is fully populated. The subpanel figure illustrates the hydrogen bonding interactions between CO_2_ and the ALF framework within the SC made possible by the four inward pointing formate hydrogens.^19^

Returning to why CO_2_ is distinct from the other adsorbates and displays a higher-than-expected KIT relative to its kinetic diameter, we draw attention to how CO_2_ binds within the SC and the strength of this interaction (Figure 5c,d). Namely, CO_2_ is unique among the adsorbates because it alone can completely span the length of the SC in the SC_*z*_ direction (subpanel of Figure 5d), engaging in four substantial hydrogen bonding interactions (2.42(1) Å).^42^ From inelastic neutron scattering (INS) and complementary DFT computation, we find that adsorbed CO_2_ blue shifts (stiffens) the important wagging formate modes centered primarily near 125 cm^–1^ (mode **3**, see Figure 3), indicating that upon CO_2_ adsorption the wagging modes are being dampened. This result potentially explains the anomalously high CO_2_ KIT (relative to kinetic diameter) as well the unanticipated peak spread in the TPD derivative for CO_2_ (three peaks were needed to fit the CO_2_ TPD instead of the anticipated two given our kinetic barrier calculations). Furthermore, what can be noted from the experimental data in Figure 5c is that, given that the small cavity cannot be completely populated (e.g., a percolation issue, see high-pressure CO_2_ isotherms Figure S10), the experimental INS spectra of CO_2_ loaded ALF is a combination of the DFT simulated bare and the CO_2_ loaded ALF spectra. From a structural perspective, the higher the KIT, the greater the TPD peak spread. The pronounced percolation issues likely all stem from the hydrogen bonding between CO_2_ and the ALF framework, which essentially “locks” the crystal structure locally, blocking apertures of diffusion and dampening framework dynamics. This is also compounded by the fact that ALF contracts as CO_2_ adsorbs into ALF (lattice parameter tracing, Figure S21).^42^

### 2.5 Application of the KITs to Separation Design

In terms of applying KITs toward mixed-gas separation design for ALF, and likely current and future ultramicroporous materials, it should be appreciated that each gas has a specific temperature regime where adsorption is maximized as a function of thermodynamics and kinetics. For ALF, this includes the low-temperature regime (<150 K) for the smaller kinetic diameter gases like He, Ne, and H_2_, and the intermediate to high-temperature range (>150 K) for larger kinetic diameter gases like O_2_, N_2_, CO_2_, C_2_H_2_, and Ar. Gas separations between the small and large gases (say the unreported separations of He vs O_2_, H_2_ vs CO_2_, or Ne vs Ar using ALF) can be achieved given the larger gases (O_2_, N_2_, CO_2_, C_2_H_2_, and Ar) can be gated absolutely by ALF’s crystal structure at lower temperatures. However, KITs become more consequential when kinetic separations of similarly sized gases are sought.

For example, the noncryogenic kinetic separation of O_2_ from N_2_ using ALF is excellent and can be achieved at ≈200 K with an Ideal Adsorbed Solution Theory (IAST) selectivity value in the range 50–125 at air-relevant concentrations and pressures.^43^ To date, this is one of the best demonstrations of O_2_ vs N_2_ filtering, especially given ALF adsorbs O_2_ instead of N_2_, amplifying its efficiency. This kinetic separation, in retrospect, is an example of how KITs can be exploited for separation design. At ≈200 K, which is near the KIT of O_2_ but 100 K lower than the KIT of N_2_, the process shows excellent performance even though the heats of adsorption of O_2_ vs N_2_ are comparable (see Figure 4a). This is because at 200 K the gating effect is leveraged to allow only O_2_ into ALF while N_2_ is kinetically gated. Alternatively, when the separation of O_2_ and N_2_ is attempted near ≈298 K (and 1 bar), closer to the lowest KIT of N_2_ and 100 K above the lowest KIT of O_2_, the idealized separation fails (IAST of ≈1).^43^ At that temperature, gating effects for both gases are eliminated, and the two gases behave similarly due to thermodynamics.

Along those lines, if we envision that we have separated O_2_ from an air stream using ALF, we would be left with predominantly (≈98%) N_2_ and a balance of Ar. With our developed understanding of separation design with ALF, it would be reasonable to infer from both thermodynamics and KIT guidance that separating N_2_ from Ar using ALF would be difficult. This can be appreciated because N_2_’s highest heat of adsorption is only slightly stronger than that of Ar, and the lowest KITs for N_2_ and Ar are approximately the same (≈310 K). This means that there is likely no temperature where kinetic gating, absolute gating, or thermodynamics could be exploited effectively to remove Ar from N_2_ with ALF.

In terms of separating CO_2_ from N_2_ using ALF,^42^ this is a delicate balance of gating and thermodynamics. As can be seen from Figure 4, CO_2_ has a strong thermodynamic driving force for adsorption into the SC due to the hydrogen bonding interactions. At ≈310 K, even though we have not yet surpassed the lowest temperature KIT of ≈336 K for CO_2_, we have overcome the thermodynamic driving force for N_2_ adsorption. This subsequently allows ALF to achieve excellent CO_2_ separation from N_2_ near room temperature, even if CO_2_ adsorption is kinetically limited. Said differently, in both the O_2_ vs N_2_ and CO_2_ vs N_2_ separations, N_2_ encounters the worst of both scenarios with ALF. When the kinetics of N_2_ adsorption become favorable near 300 K, the heat of adsorption of N_2_ into both cavities has been largely overcome and no thermodynamic driving force exists. However, though adsorption of N_2_ at 1 bar is very limited, some adsorption can occur when forced with increasing pressure (Figure S8).

The adsorption of C_2_H_2_ (for C_2_H_2_ vs CO_2_ separation) with ALF is another case of unfavorable kinetic/thermodynamic pairing.^45^ For C_2_H_2_, DFT-calculated adsorption energies indicate that the LC is the preferred cavity for adsorption, with a heat of adsorption energy comparable to that of CO_2_ in the SC_*z*_ orientation. However, the energetic barriers to enter/leave the LC are quite high (725 and 854 meV, respectively, Figure 5a), corresponding to calculated KIT values of roughly ≈390 and 460 K, respectively. The lowest KIT for C_2_H_2_, as predicted from DFT and verified from TPD, is for the SC-to-SC gating, which has a value at ≈293 K. As such, at 293 K, much like for N_2_ in ALF, the SC heat of adsorption for C_2_H_2_ has largely been overcome, preventing appreciable uptake given that the SC’s account for 75% of the cavities in ALF. As the temperature is increased further, attempting to overcome the SC-to-LC KIT at ≈390 K, adsorption of C_2_H_2_ is both low and kinetically limited.^45^ When the SC-to-LC KIT is finally surpassed near 390 K, the kinetics of adsorption are presumably fast, but any thermodynamic driving forces have been overcome. As such, ALF has record-setting CO_2_ vs C_2_H_2_ separation metrics near room temperature with an IAST CO_2_/C_2_H_2_ adsorption selectivity of ≈10^6^.

Lastly, we note that the effect of defects on an adsorbent system like ALF can have major implications on separation performance. This is because crystallographic defects would likely provide alternative routes for gases to penetrate the adsorbent, bypassing the gating effects that enable both absolute and kinetic separations. An exaggerated example of this has been shown by us with the ALF chemical congener Fe-ALF, which has approximately half of the Al^3+^ substituted with Fe^3+^.^43^ Crystallographically, introducing Fe^3+^ creates structural distortions in the ALF structure, altering the orientations (and likely vibrational behavior) of the formates that gate the cavities. Experimentally, it was observed that the adsorption kinetics with Fe-ALF with O_2_ and N_2_ were faster than unsubstituted ALF, but with a slightly diminished ability for sustained kinetic separations. For defective samples like Fe-ALF with modified gating structures, kinetic separations of similar gases would likely remain challenging but would serve as ideal adsorbents for single component gases. In our analysis of ALF, we have consistently found no evidence of defects. This detail strongly supports why our DFT predictions (which do not model defects) and KIT analysis resoundingly predict ALF’s performance.

## 3 Conclusions

In conclusion, we have shown that understanding a temperature-regulated gating material like ALF is best done through a multifaceted approach. Specifically, we have used high-quality diffraction studies to provide structural information, spectroscopic investigations to isolate specific vibrations of interest, and adsorptive studies to identify temperatures of peak adsorption for each adsorbate. When combined, these probes paint a comprehensive picture of a temperature-regulated adsorbent such as ALF. However, the implications of our theoretical NEB calculations reveal that when select experimental data is combined with targeted computation, accurate estimates of real-world performance can be made without exhaustive experimentation. This is done through KIT analysis.

Our developed KIT analysis shows how to isolate and predict the important tipping point temperatures where the kinetics of diffusion and the thermodynamics of adsorption exchange significance for adsorbates in ultramicroporous materials. Previously, understanding the origin of such behavior in ultramicroporous materials and predicting such temperatures for untested adsorbates have been difficult. Given the growing number of materials like ALF that show “inverse-selectivity” for adsorbates that leverage temperature to achieve kinetic separations, KIT analysis should hone future material characterization and separation design for this important material class.

Furthermore, our work serves as a guide to researchers who suspect they have found a temperature-regulated gating adsorbent or those working in the greater field of ultramicroporous materials. It is our suspicion that there are likely ultramicroporous materials that have been previously deemed nonporous, but which may in fact have just been temperature-regulated gating adsorbents that were tested at a nonideal temperature with a nonideal adsorbate. As such, it is our recommendation that to fully establish an ultramicroporous material’s porosity, experiments across larger thermal windows with more chemically varied gases should be done routinely.

## 4 Experimental Section

### 4.1 Reflux Synthesis of Al­(HCOO)_3_

The as-made Al­(HCOO)_3_ was synthesized using the reported procedure.^42,62^ In a typical synthesis, formic acid (100 mL) and aluminum hydroxide (1.2 g, 0.015 mol) were refluxed in a 250 mL three-necked round-bottomed flask at 100 °C (373 K) for 48 h. After completing the reaction, excess formic acid was extracted by centrifugation, and the white solid was rinsed with a copious amount of ethanol and separated using vacuum filtration. The air-dried sample gave a yield of 95% white solid product of Al­(HCOO)_3_(CO_2_)_0.75_(H_2_O)_0.25_(HCOOH)_0.25_/(guest included/as-made Al­(HCOO)_3_).

### 4.2 Activation of As-Made Al­(HCOO)_3_ to ALF

The air-dried, as-made Al­(HCOO)_3_(CO_2_)_0.75_(H_2_O)_0.25_(HCOOH)_0.25_ was activated according to the following procedure: heated at 150 °C (423 K) for 24 h under high vacuum (1 × 10^–4^ mmHg). Alternatively, the sample can be heated in air/ambient conditions at 180 °C (453 K) for 24 h, yielding quantitative amounts of the guest-free Al­(HCOO)_3_ (ALF).

### 4.3 Neutron Powder Diffraction (NPD)

Neutron diffraction measurements were performed on a 1.23 g activated sample of ALF powder at the National Institute of Standards and Technology Center for Neutron Research (NCNR). Data were collected at the high-resolution neutron powder diffractometer, BT-1, utilizing a Ge(311) monochromator with an in-pile 60′ collimator, corresponding to a neutron wavelength of 2.079 Å. The sample was loaded into a vanadium sample can in a He environment glovebox and sealed with a soldered lead O-ring onto a copper heating block containing a valved outlet for gas loading. After mounting the sample onto a bottom-loaded closed-cycle refrigerator (CCR), the sample was reactivated at elevated temperatures under vacuum to remove possible residual helium. The sample was then cooled and measured at various temperatures for sufficient time to be able to perform high-quality Rietveld refinements, or with 1-h scans to obtain unit cell values on heating.

### 4.4 Synchrotron X-ray Powder Diffraction (APS and XLS-II)

High-resolution synchrotron X-ray powder diffraction data was collected at Beamline 17-BM at the Advanced Photon Source (APS) at Argonne National Laboratory. The temperature of the capillary sample was achieved using an Oxford Cryosystems Cryostream 800. Scattered intensity was measured by a PerkinElmer amorphous-Si flat panel detector. The wavelength for the measurements was 0.24101 Å. Powder samples of previously activated ALF were loaded into 1.0 mm quartz capillaries in air. The capillaries were then attached to custom valve-based dosing sample holders connected to a known volume gas dosing manifold with vacuum pump and reactivated under dynamic vacuum at 360 K until the pattern recorded stopped changing (≈10 min).

High-resolution synchrotron X-ray powder diffraction data was also collected at the XPD Beamline 28-ID-2 at XLS-II at Brookhaven National Laboratory. The temperature of the capillary sample was achieved using an Oxford Cryosystems Cryostream 800. Scattered intensity was measured by a PerkinElmer amorphous-Si flat panel detector. The wavelength for the measurements was 0.1827 Å. Powder samples of previously activated ALF were loaded into 1.0 mm quartz capillaries in air. The capillaries were then attached to custom valve-based dosing sample holders connected to a known volume gas dosing manifold with vacuum pump and reactivated under dynamic vacuum at 360 K until the pattern recorded stopped changing (≈10 min).

For gas dosing diffraction experiments run with a similar procedure to TPD experiments, the following procedure was utilized using our known volume valve-based sample dosing system. A sample of ALF (≈20 mg) was loaded with ≈1 bar (1 bar = 100 kPa) of pressure (CO_2_ or O_2_) at 330 K for ≈30 min. The sample was then cooled to ≈200 K for CO_2_ and evacuated, or to 100 K for O_2_ and evacuated. The cooling rate was the maximum rate afforded by the cryostream, ≈15 K per min. The sample holder valve was closed so the vacuum was static, and volume of the system was reduced to only the capillary, then the sample was heated at 4 K per minute. The capillaries used for the experiment were wide funneled quartz capillaries with 10-μm width walls and 1 mm outer diameters. For separate high-pressure measurements taken at the APS, a syringe pump was used to achieve pressures between 0 and 40 bar. Data was monitored until a time where data appeared to stop changing, and then pressure was increased.

### 4.5 Powder Diffraction Rietveld Refinement Procedure

Diffraction data was analyzed using the TOPAS 6 software suite.^63^ Rigid bodies were employed for certain gas molecules where necessary.

### 4.6 Gas Isotherms

Prior to sorption studies, ALF samples were activated under a dynamic vacuum (≈10^–3^ mmHg) for 12 h at 110 °C (383 K). The sample masses tested were consistently ≈300 mg for ALF. After each measurement, the sample was heated to 323 K and pumped for several hours.

Volumetric gas adsorption measurements were performed using a custom developed fully computer-controlled Sieverts apparatus as discussed previously.^44,64,65^ Briefly, the fully computer-controlled Sievert apparatus operates in a sample temperature range of 20 to 500 K and a pressure range of 0 to 100 bar (1 bar = 100 kPa). In the volumetric method, gas is admitted from a dosing cell with known volume to the sample cell in a controlled manner; the gas pressure and temperature are controlled and recorded.

Some unique features of the setup are as follows; the instrument has multiple gas inlets for various gases of interest, which enables pore volume and surface measurements followed by He-cold volume determination and finally the gas adsorption measurements without needing to move the sample from the cell. Two high precision pressure gauges with parts-per-billion resolution and typical accuracy of 0.05% (20 psia (≈1.38 bar) and 500 psia (≈34.47 bar), respectively) were used to precisely measure the pressure. For isotherm measurements below room temperature, the sample temperature was controlled using a closed-cycle refrigerator (CCR). The difference between the actual sample temperature and the control set-point is within 1 K over the whole operating temperature range. The connection between the sample cell and the dose cell is through 1/8 in (0.3175 cm) capillary high-pressure tubing, which provided a sharp temperature interface between the sample temperature and the dose temperature (i.e., room temperature). The cold volumes for the empty cell were determined using He as a function of pressure at every temperature before the real sample measurement and were used to calculate the sample adsorption.

Since the adsorbed amount is deducted from the raw P–V–T data using a real gas equation of state, a critically important issue is the accuracy of the chosen equation of state (EOS) in terms of describing the real gas behavior within the desired temperature and pressure range. Using an empty cell as a reference, we found that the MBWR EOS best describes the real gas behavior of gases tested. Therefore, in all our isotherm data reduction, the NIST MBWR EOS is used. [NIST Standard Reference Database 23: NIST Reference Fluid Thermodynamic and Transport Properties Database].

### 4.7 Temperature-Programmed Desorption Studies

The Temperature-Programmed Desorption (TPD) studies conducted here offer an alternative method for monitoring desorption, as the setup does not utilize a mass spectrometer to track desorbing gas molecules. Instead, desorption is monitored by measuring the increase in pressure within the system, given that only one gas is tested at a time.

The sample is initially heated to 400 K under vacuum, after which the dosing gas is introduced at ≈10 bar pressure (except for C_2_H_2_, which was ≈2 bar.) While the sample remains under this applied gas pressure, it is gradually cooled over 6 h to the liquefaction temperature of the dosing gas. At this temperature, the system is pumped down until the base vacuum pressure is reached. Once this condition is achieved, the pump is closed, and a temperature ramp is initiated, heating the sample at a rate of 5 K/min. During the temperature ramp, pressure is continuously monitored and recorded as a function of temperature. Given that the dosing volume and temperature are known, the amount of desorbed gas from the sample can be calculated as a function of temperature. Additionally, by taking the derivative of the desorbed gas with respect to temperature, we can determine the KITs, as shown in the main text.

### 4.8 Kinetic Adsorption Studies

Adsorption kinetics measurements were conducted using the same computer-controlled Sievert apparatus described above. Briefly, the sample is first equilibrated at a given temperature, and the dosing volume is filled with the selected gas at approximately ≈8 bar. The sample valve is then opened, allowing the gas in the dosing volume to be exposed to the sample, while the pressure drop is monitored as the gas adsorbs onto the sample over time. At any given time, the amount of gas adsorbed is calculated based on the pressure data. These kinetic data are presented in Figures S11–S17. By repeating these kinetic measurements at different temperatures, we determine the adsorption kinetics as a function of temperature. From Arrhenius fitting of the kinetic data, we obtain the activation barrier (*E*_a_) for adsorption of the given gas.

### 4.9 Raman Data Collection

A pressed powder pellet of ALF was mounted inside an optical cryostat under a partial helium atmosphere. The temperature in the sample space can be varied from 2 to 300 K. A 515 nm laser line from an Ar ion laser was used to excite the sample, through a cryogenic 50× objective. The laser power was kept at 2.5 mW, measured before the optical window of the cryostat. The laser spot size was approximately several microns due to the roughness of the pellet. The backscattered Raman beam was collected by the same objective and sent to a triple grating Raman spectrometer operating in triple-subtractive mode, which rejected the elastic light (Rayleigh scattering) and sent the Raman scattering to a liquid nitrogen cooled CCD. The Raman beam could be manipulated by several polarization optics to select the two measurement polarization configurations, VV and VH. Data presented and fit is from VV configuration.

### 4.10 Raman Peak Fitting

Peak fitting of variable temperature Raman spectra of ALF was performed using the multipeak package within the Igor 7 software suite. Spectra taken up to 150 K were fit, as at temperatures above 150 K, the wagging mode areas (mode areas **2** and **3**, see main text Figure 3) overlapped to a point where separate peak fitting and assignment became inconclusive. Mode identities for classification were assigned via DFT-calculated positions from a neutron powder diffraction .cif file at 15 K (Table S5).

We note that in our DFT-calculated Raman spectra, certain modes are calculated as degenerate, but experimentally, are found to be distinct. The mode area at ≈113 cm^–1^ (labeled as mode area **2**) is one such cluster of modes. To account for this, certain mode areas were conservatively modeled as one distinct spectral area with a cumulative spectral weight to reduce ambiguity.

### 4.11 Inelastic Neutron Scattering (INS)

INS spectra were recorded on the VISION spectrometer at Spallation Neutron Source, Oak Ridge National Laboratory (ORNL). VISION is an indirect geometry crystal analyzer instrument that provides a wide dynamic range with high resolution. The ALF sample was loaded into a cylindrical aluminum sample container with an indium vacuum seal and then connected to a gas handling system. The sample was degassed at ≈10^–7^ mbar at 360 K prior to experimentation to remove trace guest molecules. The temperature during data collection was controlled using a closed-cycle refrigerator (CCR) cryostat. INS spectra of bare ALF were taken up to 200 K. Specific to data with ALF loaded with CO_2_, the loading was performed near room temperature with a stoichiometric amount of CO_2_. The temperature was then reduced to 5 K to perform the scattering measurements and to achieve minimum thermal motion for the framework host and adsorbed CO_2_ molecules.

### 4.12 Density Functional Theory Modeling of Inelastic Neutron Scattering and Raman Spectra

INS: Modeling by Density Functional Theory (DFT) of the bare and CO_2_-loaded ALF was performed using the Vienna Ab initio Simulation Package (VASP).^66^ The calculation used Projector Augmented Wave (PAW) method^67,68^ to describe the effects of core electrons. The energy cutoff was 800 eV for the plane-wave basis of the valence electrons. The lattice parameters (*a* ≈ 11.4 Å) and atomic coordinates determined by the current work were used as the initial structure. The electronic structure was calculated on a 5 × 5 × 5 Monkhorst–Pack mesh. The total energy tolerance for electronic energy minimization was 10^–8^ eV, and for structure optimization, it was 10^–7^ eV. The maximum interatomic force after relaxation was below 0.001 eV/Å. The optB86b-vdW functional^69,70^ with dispersion corrections was applied. The vibrational eigenfrequencies and modes were then calculated using the finite displacement method and phonopy.^71^ The OClimax software^72^ was used to convert the DFT-calculated phonon results to the simulated INS spectra.

Raman: The vibrational spectra of ALF were simulated using density functional theory (DFT) as implemented in the all-electron code CRYSTAL17,^73,74^ where the crystalline wave functions are expanded as a linear combination of atomic orbitals and further expanded by a consistent triple-ζ and polarization basis-set.^75^ The DFT total energy during the geometry relaxation and in the Raman calculations was converged within ≈10^–10^ a.u. and integrated over a well-converged and symmetrized 6 × 6 × 6 k-point mesh. Forces were converged using the default convergence criteria. Whenever possible, the internal symmetry was maintained. The unknown exchange–correlation contribution to the total energy was approximated by a hybrid functional that combines the PBE-Generalized gradient functional by Perdew, Burke, and Ernzerhof with 25% exact exchange.^69,76^

### 4.13 Quasielastic Neutron Scattering (QENS)

QENS measurements were carried out using the near-backscattering spectrometer, BASIS, at the Spallation Neutron Source (SNS) at Oak Ridge National Laboratory (ORNL). An incident neutron bandwidth centered at 6.4 Å was used, covering an energy window of ±120 μeV with an energy resolution of 3.6 μeV (full width at half-maximum). The sample was prepared using a thin-walled aluminum annular can with 0.1 mm thickness of sample to minimize the multiple scattering contribution. The ALF sample can was placed in a top-loading closed-cycle refrigerator, and the measurements were carried out between 100 and 350 K. Data was analyzed using the DAVE software suite.^77^ Mean squared displacement values as a function of *Q* were calculated for each QENS temperature data set using the equation:

1 $$<u^{2}>\left(Q\right)=\left(-\frac{3}{2}\right)\;\log\left(I\right)$$

### 4.14 Density Functional Theory Calculations for Binding Energies and Kinetic Barriers

Density functional theory (DFT) calculations were performed using the Vienna *ab initio* simulation package (VASP) version 5.4.1.^66,78,79^ Projector augmented wave (PAW) potentials were used for all atoms^68^ using the PBE functional.^69^ A 520 eV kinetic energy cutoff was used in the treatment of the valence electrons with a self-consistent field (SCF) energy cutoff of 1.0 × 10^–7^ eV. The geometry relaxation steps were assumed to converge following an energy difference of 5.0 × 10^–5^ eV between two ionic steps. The precision of the calculations was set to accurate using the *PREC* keyword. A 2 × 2 × 3 Monkhorst–Pack k-point mesh was used. Dispersion corrections are included using the method of Grimme with Beck-Johnson damping (D3BJ).^80,81^ Kinetic barriers of gas migration were calculated using the nudged elastic band (NEB) as implemented in the VASP transition state tools (VTST).^82,83^

## Supplementary Material





## Notes

ALF Formate Wagging (MP4)
The SI contains supplementary text discussing gas studies and DFT calculations. It also contains supporting figures and supporting tables of information regarding diffraction, neutron scattering, spectroscopy, gas studies, and computational calculations (PDF)
