# Supplementary material for: Temperature-Regulated Gating Enables Gas Separations in Ultramicroporous Aluminum Formate, ALF
Source: Chem Mater. 2025 Sep 2;37(18):7102–14. doi: 10.1021/acs.chemmater.5c01157 (PMC12461827; doi:10.1021/acs.chemmater.5c01157)
Supplement: Supplementary file 2 [file cm5c01157_si_002.pdf]

Supporting Information:

Temperature-Regulated Gating Enables Gas Separations in Ultramicroporous Aluminum Formate, ALF

Hayden A. Evans*,[1] Taner Yildirim,[1] Gavin A. McCarver,[1] Thuc T. Mai,[2] Yongqiang Cheng,[3] Zeyu Deng,[4] Ryan A. Klein,[1,5] Dan Zhao,[6] Pieremanuele Canepa,[7] Angela R. Hight Walker,[2] Anthony K. Cheetham,[4,8] Craig. M. Brown[1]

1. Center for Neutron Research, National Institute of Standards and Technology, Gaithersburg, Maryland 20899, USA

2. Physical Measurement Laboratory, National Institute of Standards and Technology; Gaithersburg, Maryland 20899, USA

3. Neutron Scattering Division, Oak Ridge National Laboratory, Oak Ridge, Tennessee 37830, USA

4. Department of Materials Science and Engineering National University of Singapore, Singapore 117585, Singapore

5. Material, Chemical, and Computational Sciences Directorate, National Renewable Energy Laboratory, Golden, Colorado 80401, USA

6. Department of Chemical and Biomolecular Engineering, National University of Singapore, Singapore 117585, Singapore

7. Department of Electrical & Computer Engineering, University of Houston, Houston, Texas 77204, USA

8. Materials Research Laboratory, University of California; Santa Barbara, California 93106, USA

***Corresponding Author: hayden.evans@nist.gov**

National Institute of Standards and Technology Adsorbent Material Database

This material and information about its reported isotherm performance can be found in the NIST Registry of Adsorbent Materials as aluminum formate / ALF.

https://adsorption.nist.gov/matdb/index.php#home

Table of Contents

Supplemental Discussion **5**

Kinetic Adsorption Studies Below Lowest Temperature KITs **5**

Figure S1: Experimental activation energies vs SC-to-SC KITs or DFT kinetic barriers **5**

In-situ rapid Acquisition Diffraction Experiments Mimicking TPD of CO_2 and O_2 in ALF **7**

Figure S2: TPD results for CO_2 desorption alongside sequential Rietveld refinement results from in-situ diffraction experiments mimicking TPD **7**

Figure S3: TPD results for O_2 desorption alongside sequential Rietveld refinement results from in-situ diffraction experiments mimicking TPD **8**

Treatment of rotation in DFT calculations for polyatomic molecules **10**

Supplemental Figures and Tables **11**

Adsorption/Desorption Isotherms **11**

Figure S4: He excess isotherms between 70 K and 120 K **11**

Figure S5: Ne excess isotherms between 120 K and 165 K **12**

Figure S6: Ar excess isotherms between 298 K and 393 K **13**

Figure S7: H_2 excess and total isotherms between 77 K and 296 K **14**

Figure S8: N_2 excess isotherms between 340 K and 393 K **15**

Figure S9: O_2 excess isotherms between 125 K and 297 K **16**

Figure S10: CO_2 excess isotherms between 340 K and 393 K **17**

Kinetic Adsorption Profiles and Fitting **18**

Figure S11: He Kinetic adsorption profile and fitting **18**

Figure S12: Ne Kinetic adsorption profile and fitting **19**

Figure S13: Ar Kinetic adsorption profile and fitting **20**

Figure S14: H_2 Kinetic adsorption profile and fitting **21**

Figure S15: N_2 Kinetic adsorption profile and fitting **22**

Figure S16: O_2 Kinetic adsorption profile and fitting 23

Figure S17: CO_2 Kinetic adsorption profile and fitting 24

Table S1: Avrami fitting results for kinetic adsorption studies for gases with ALF 25

KIT Fitting Results........ 26

Table S2: Experimental KITs resolved from TPD fitting 25

Diffraction........ 27

Figure S18: Select Rietveld refinement results of neutron and synchrotron X-ray data of bare ALF between 10 K and 380 K........ 27

Figure S19: A representative sequential Rietveld refinement result from results shown in Figure SX of synchrotron X-ray........ 28

Figure S20: A representative sequential Rietveld refinement result from results shown in Figure SX of synchrotron X-ray data taken of ALF loaded with CO_2 and heated, replicating TPD experiments........ 29

Figure S21 In-situ rapid acquisition X-ray diffraction with ALF at 270 K during CO_2 adsorption up to 39 bar 30

Thermal Expansion 31

Figure S22: Calculated thermal expansion coefficients for ALF........ 31

Figure S23: Experimental thermal expansion coefficients obtained from neutron and X-ray diffraction 32

Raman Fitting........ 33

Figure S24:: Raman fitting performed on peak areas **1-4** between 60 cm−1 and 200 cm^{-1}. 33

Figure S25: Raman fitting performed on peak areas **5-7** between 260 cm−1 and 400 cm^{-1}. 34

Neutron Spectroscopy Fitting 35

Figure S26: Inelastic neutron scattering spectra of bare ALF between 5 K and 200 K.. 36

Figure S27: Quasielastic Neutron Scattering results on bare ALF graphed as a function of mean squared displacement versus temperature........ 36

Density Functional Theory........ 37

Figure S28: Atomistic view of the intrapore rotation of CO_2 in the SC of ALF... 37

Figures S29: Energy diagrams for the intrapore rotation of H_2, O_2, N_2, CO_2, and C_2H_2

in the SC of ALF........ 38

Figure S30: Energies of gas migration from SC-to-SC (circles) and SC-to-LC (squares) for the 8 different adsorbates........ 39

Figure S31: Graph illustrating the TPD derived KITs vs corresponding SC-to-SC and LC-to-SC barriers. 40

Table S3: DFT calculated kinetic barriers between cavities (meV)........ 41

Table S4: DFT calculated binding energies for each adsorbate in the three SC orientations and LC (meV). **42**

Table S5: DFT calculated kinetic barriers between cavities (kJ/mol) **43**

Table S6: DFT calculated binding energies for each adsorbate in the three SC orientations and LC (kJ/mol). **44**

Table S7: Simulated Raman modes and their intensities. **45**

Supporting Information References **46**

Supplemental Discussion

<u>Kinetic Adsorption Studies Below Lowest Temperature KITs</u>

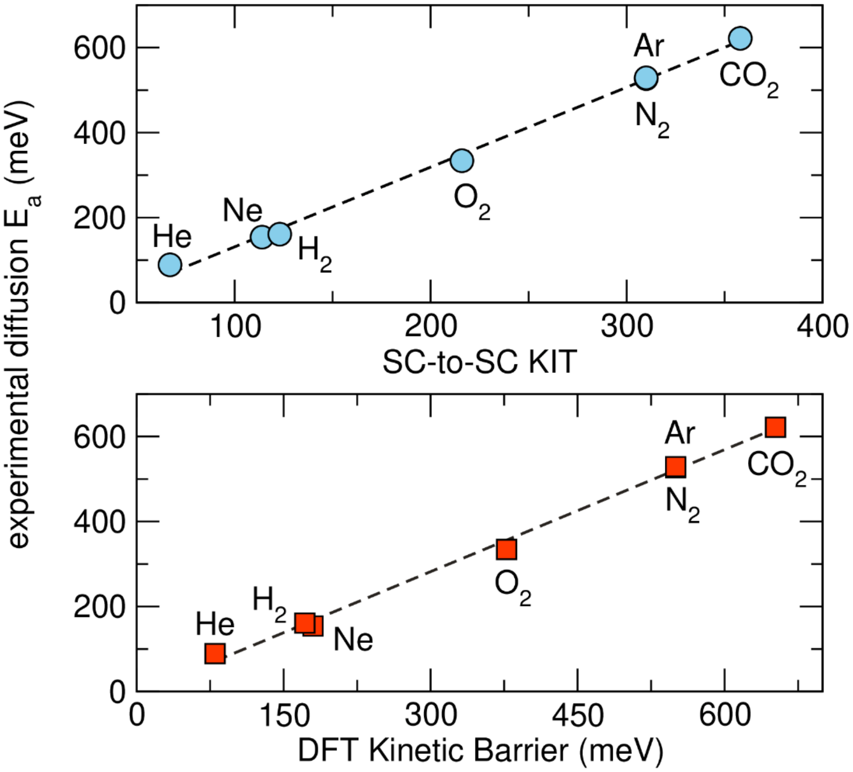

Figure S1: Graphs of activation energies derived from kinetic adsorption experiments performed for all gases (except C_2H_2) vs SC-to-SC KITs or DFT calculated kinetic barriers. (top) SC-to-SC KIT temperatures vs kinetic adsorption activation energies (R^2 = 0.9969). (bottom) DFT calculated kinetic barriers (meV) vs kinetic adsorption activation energies (R^2 = 0.9934). These plots confirm the trend illustrated in the main text between TPD derived KITs and DFT calculated kinetic barriers, highlighting the efficiency of utilizing only TPD vs DFT calculated kinetic barriers for predictive purposes instead of experimental methods alone. The values used in this graph can be found in Tables S1 – S3. C_2H_2 was not tested because each other gas was tested with ≈ 8 bar of starting pressure, and C_2H_2 could not be brought to that pressure without fear of explosion.

As shown in the main text, Kinetic Inflection Temperatures (KITs) were established for each of the tested gases and correspond to specific kinetic migration barriers within the crystal structure of ALF that need to be overcome for efficient diffusion. We have documented in this work and previous publications, [1–4] how above and below these temperatures, minimal or pronounced isotherm hysteresis can exist for each adsorbate (Figures S4 – S10). In the main text of this publication, it was shown that the SC-to-SC kinetic migration barrier proves to be the most impactful barrier governing efficient diffusion. From DFT calculations, the SC-to-SC barrier was consistently resolved as corresponding to the lowest temperature KIT for all adsorbates (except for CO_2). The significance of the SC-to-SC KIT is as a rate limiting step for efficient diffusion, which is likely convolved with the multiplicity differences between the cavities, as the ratio of SCs to LCs is 3 to 1 in ALF. Given the kinetic significance of these temperatures, further kinetic

adsorption studies were performed below the lowest temperature KITs for all gases (except C_2H_2 given safety concerns) to further probe these effects.

In our previous report on the non-cryogenic kinetic separation of O_2 from N_2 using ALF,[2] we demonstrated that the kinetic adsorption scans of O_2 and N_2 were best fit when using an empirical formula (Equation 1) instead of pseudo-first/second order equations. The empirical equation was:

$$\boldsymbol{q_t = q_m * \left(1 - e^{-(kt)^{\frac{1}{2}}}\right)} \qquad (2)$$

Where q_t = observed adsorption, q_m = adsorption maximum, k = rate constant, t = time. At publication,[2] the inclusion of the 0.5 exponent had limited physical significance, but upon further consideration, equation (1) is now understood to be a form of the Avrami equation,[5] where q_m is a scaling variable, and the independent Avrami exponent (n) is fixed at 0.5.

For background, the Johnson–Mehl–Avrami–Kolmogorov formalization, often referred to as the Avrami equation, was originally developed to describe the progress of phase transformations in material systems. The equation has also been routinely applied to other phenomena but remains most useful for describing diffusion-controlled growth of materials. This includes diffusion of gas molecules into solids. Interestingly, the 0.5 exponent in the Avrami equation does have a physical meaning and provides further clarity for understanding the diffusion of gas molecules through the ALF system. Specifically, the ALF system, which can be described as a diffusion-controlled growth system with instantaneous nucleation, the 0.5 exponent indicates needle-like diffusion.[5]Physically, this needle-like diffusion describes captures how gas molecules can only propagate one molecule at a time from one cavity to the next in ALF, moving along 2D channels. The results of our Avrami kinetic fitting and subsequent activation energies are shown in Figures S11 – S17 and Table S1.

From the kinetic studies below the KITs for the gases, we can confirm the trends seen from DFT calculation to experimental results. Specifically, a linear trend not only exists for the calculated barriers vs KITs (main text, Figure 4), but also KITs vs kinetic adsorption study activation energies, and calculated kinetic barriers vs kinetic adsorption study activation energies (Figure S1).

In-Situ Rapid Acquisition Diffraction Experiments Mimicking TPD of CO_2 and O_2 in ALF

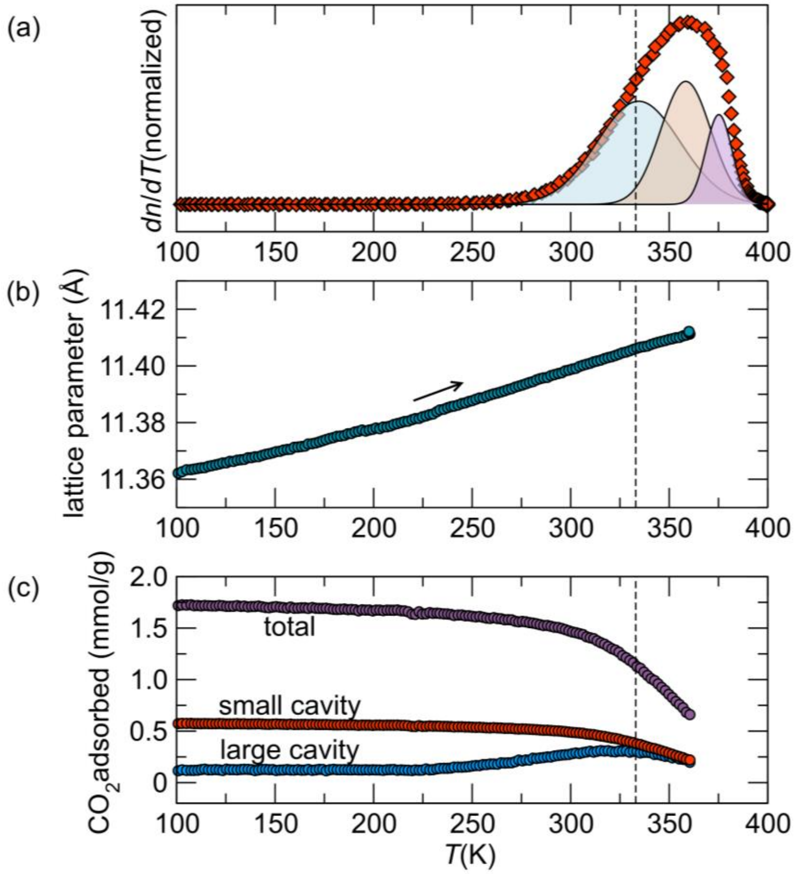

Figure S2 TPD results for CO_2 desorption alongside sequential Rietveld refinement results from in-situ diffraction experiments mimicking TPD experimental conditions [XPD, BNL]. (a) Replotting of the TPD results and derivative fitting shown in the main text (Figure 2) for ease of comparison with diffraction results. (b) Unit-cell change of ALF upon heating and desorption of CO_2. and (c) Refined occupations of adsorbed CO_2 in ALF (converted to mmol/g) of ALF at every temperature. Small cavity (SC) and large cavity (LC) refer to the occupancies per cavity, and given there are three times as many SCs as LCs, the total population is equal to (3 × SC) + LC. The procedure for the diffraction experiment is described in the experimental section. Error values (1 sigma) for data points are smaller than the symbols used.

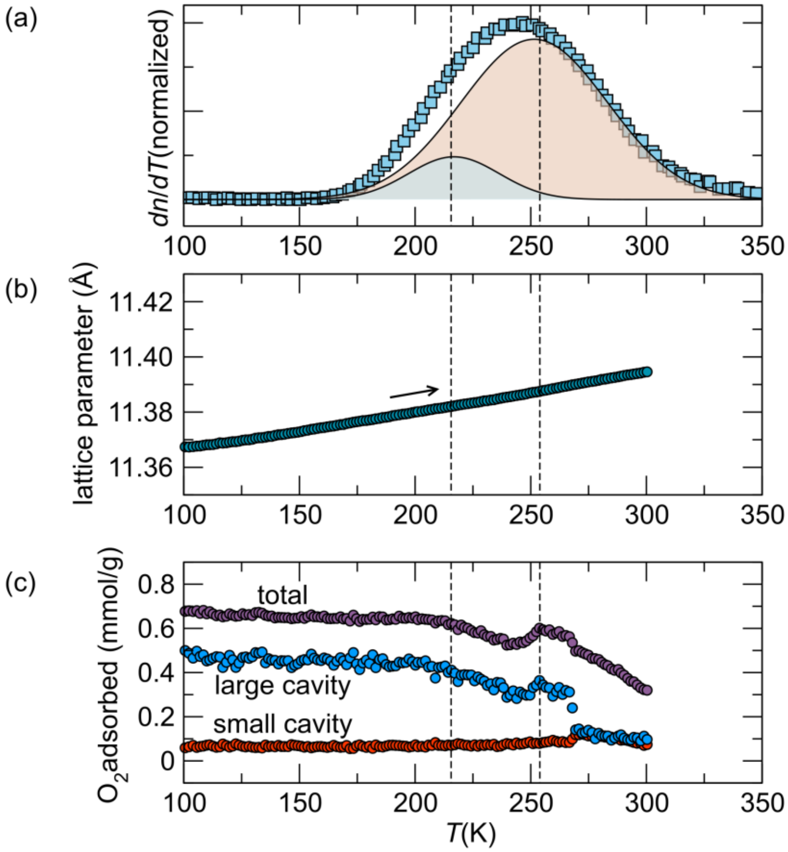

Figure S3 TPD results for O_2 desorption alongside sequential Rietveld refinement results from in-situ diffraction experiments mimicking TPD conditions [XPD, BNL]. (a) replotting of the O_2 TPD results and derivative fitting shown in the main text (Figure 2). (b) Unit cell change of ALF upon heating and desorption of O_2. and (c) Refined occupations of adsorbed O_2 in ALF (converted to mmol/g) of ALF at every temperature. The dashed lines denote the KIT centers to illustrate the changes observed in the diffraction experiment. The procedure for the experiment is described in the experimental section. Error values (1 sigma) for data points are smaller than the symbols used.

It was shown in the main text via adsorption and DFT computational studies that the KITs observed from TPD measurements correspond to when specific pathways in ALF become accessible for adsorbates. These pathways are the SC-to-SC, SC-to-LC, and LC-to-SC pathways. It was further shown how these pathways are defined by the thermally activated dynamics of the formate ligands. The wagging motion of the formates specifically, given their positioning relative to the diffusion pathways, is the dominant cause of the gating effect observed in ALF.

In adsorption studies, the gating effect can be noted as hysteresis between desorption and adsorption isotherms for an adsorbate. Specifically, if adsorption is attempted at temperatures which are too cool relative to lowest temperature KIT for an adsorbate, kinetic gating (slowed diffusion) or even absolute gating (little to no diffusion) can occur. If diffusion of an adsorbate into a cavity into ALF can occur, regardless of kinetics, adsorption is subject to thermodynamics. This thermodynamic driving force ultimately dictates which of the two cavities is most likely to populate at a given temperature/pressure if

both can be accessed. To attempt to observe these convolved effects of kinetics and thermodynamics more directly, we employed in-situ rapid acquisition X-ray diffraction to monitor CO_2 and O_2 desorption under similar conditions to TPD measurements.

Figures S2 and S3 shows Rietveld refinement results from rapid acquisition in-situ synchrotron diffraction experiments [XPD, BNL] on ALF as CO_2 and O_2 is desorbed upon heating. CO_2 and O_2 were chosen for these experiments because both gases have favorable X-ray cross sections, as well as relatively high adsorption in ALF within cryostream accessible temperatures. The full experimental procedure is described in the diffraction experimental section but are analogous to the TPD experiments shown in Figure 2. For easy comparison, the TPD results from Figure 2 have been plotted alongside the diffraction results for their respective gas.

Figure S2a shows the CO_2 TPD results from main text Figure 2, alongside the Rietveld Refinement results for the desorption diffraction experiments done with CO_2 (Figure S2b and S2c.) What can be seen in Figure S2b is that upon heating from 100 K, the unit cell of ALF increases monotonically from 100 K to 350 K. In Figure S2c, the Rietveld refinement results of the total and per cavity amount (mmol/g) of CO_2 adsorbed are shown, illustrating how the system changes as temperature is increased. Note that the total filling of the ALF system is equal to (3 × SC population) + (1 × LC population). We can see in Figure S2c that the total CO_2 population within ALF after cooling is ≈ 1.75 mmol/g CO_2. However, even though the sample was filled at ≈ 330 K, where previous at that temperature show both cavities populated with CO_2, at 100 K, the population of CO_2 is almost entirely localized to the SCs. This verifies that upon cooling, the driving force for adsorption into the SCs is quite strong as DFT calculated binding energies suggest. When heating from 100 K, this SC cavity preference persists until ≈ 225 K, until the SC population decreases, and the large cavity population increases. This change in population also coincides with gradual desorption of the system. This SC-LC population equalization continues until ≈ 330 K, wherein the total amount of adsorbed CO_2 in ALF drops and the populations between the two cavities on a per cavity basis are near identical. As each of the KITs identified have a distribution range, this experiment shows the effect of the overlapping distributions of the KITS, and the effect of increasing temperature as desorption occurs.

Figure 5c – 5d illustrates the results of the comparable experiment with O_2 and ALF. As can be seen, the unit cell also monotonically increases between 100 K and 300 K. However, contrasting the CO_2 experiment, between 100 K and ≈ 200 K, the population of adsorbed O_2 is almost entirely within the LCs, which is supported by DFT calculations.[2] The total population of O_2 does not begin to change in quantity until temperature exceeds ≈ 200 K. Once 200 K is surpassed, the population of the LC begins to decrease, alongside desorption, until the two cavities reach occupation unity near ≈ 260 K after a precipitous drop and fluctuation at ≈ 250 K. The KIT peaks found in the O_2 TPD desorption align with events for population changes in the diffraction experiment.

The diffraction experiments contain two important conclusions. The first is that if an adsorbate has been previously adsorbed into ALF at elevated temperatures, and the temperature of the sample is brought to temperatures well below that adsorbates KIT, the adsorbate trapped within ALF will migrate and populate the cavity of thermodynamic preference (given cavity availability/accessibility). The second conclusion is that if a sample with trapped adsorbate is heated, when the sample approaches a KIT of that specific adsorbate, the population between the cavities will begin changing, drifting to reflect the favorable thermodynamics of adsorption in one of the cavities.

These observations further contextualize how ALF, and likely other temperature regulated gating adsorbents, are a delicate balance between kinetic barriers and thermodynamics driving forces. Depending

on the temperature and pressure a material system is placed, gas trapping or gas separations of choice can be engaged when KITs and kinetic barriers are understood.

Treatment of Rotation in DFT Calculations for Polyatomic Molecules

To accurately compute the kinetic barriers of gas migration for polyatomic molecules, the following general procedure was employed for each adsorbate (H_2, N_2, O_2, CO_2, C_2H_2):

1. **Thermodynamic Binding Energies** – Binding energies were calculated for three molecular orientations within the small cavity (SC_X, SC_Y, SC_Z) and three molecular orientations within the large cavity (LC_X, LC_Y, LC_Z). Due to the isotropic nature of the LC, the latter orientations were often similar. The reference energy ("zero") for each adsorbate was defined as its most energetically stable orientation. For instance, in the case of CO_2 within the SC, this corresponds to the SC_Z orientation, where four hydrogen-bonding interactions stabilize the molecule.
2. **Starting and Ending Points** – The most stable orientations in adjacent cavities were used as the initial and final states for the nudged elastic band (NEB) calculations. A simple linear interpolation was employed to generate intermediate coordinates along the gas migration pathway.
3. **Energy Minimization** – During NEB calculations, the energy minimization algorithm identified the optimal orientation of the adsorbate at each step to minimize system forces. This necessitated both translational and rotational adjustments to connect the initial and final orientations. For diatomic adsorbates, translation and rotation occurred in tandem, yielding smooth, Gaussian-like diffusion energy profiles. In contrast, for CO_2 and C_2H_2, the greater molecular length led to sharper transitions between predominantly translational and predominantly rotational movements, resulting in more complex energy profiles.

Figures S29 and S30 illustrate the determination of kinetic barriers (activation energies) for gas migration explicitly accounting for molecular rotation and highlight the differences in pathway energetics.

Supplemental Figures and Tables

<u>Adsorption/Desorption Isotherms</u>

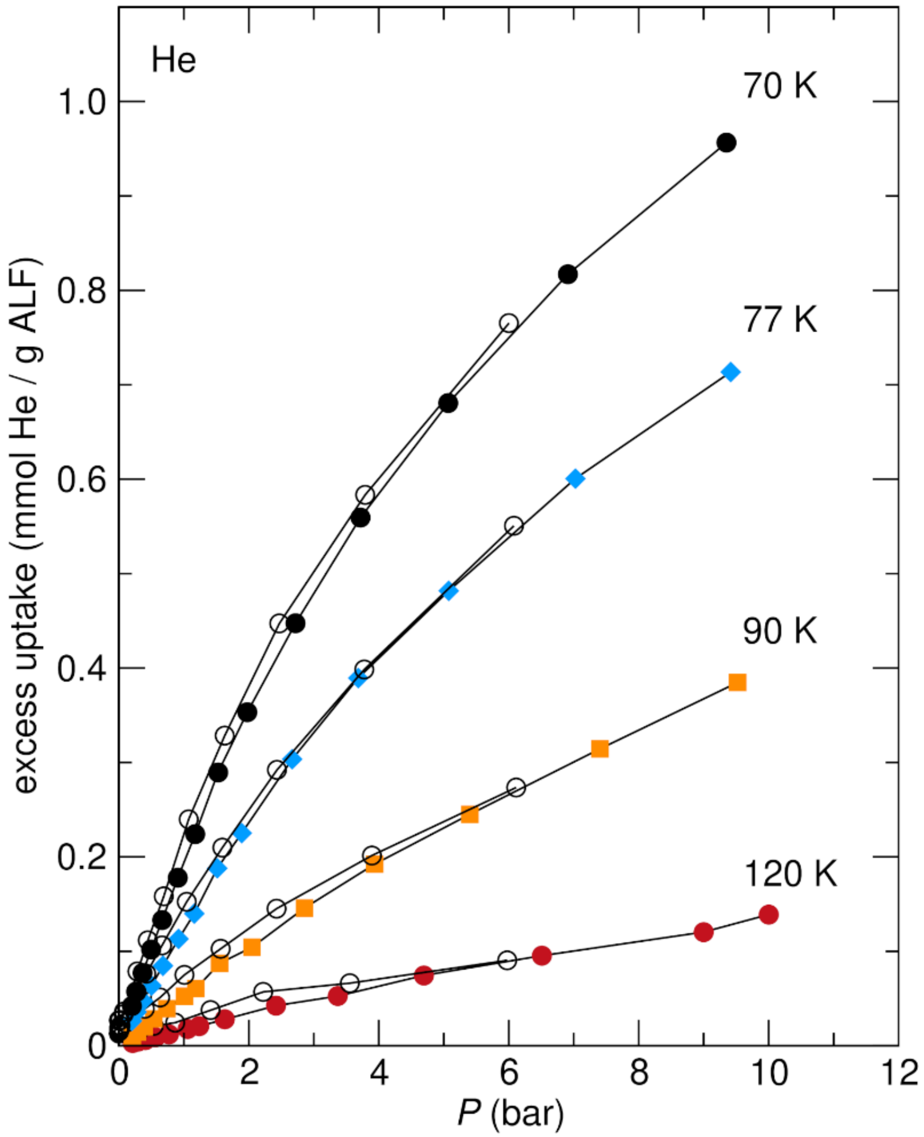

Figure S4: He excess isotherms between 70 K and 120 K. Closed symbols denote adsorption isotherms, open symbols denote desorption isotherms for each given temperature.

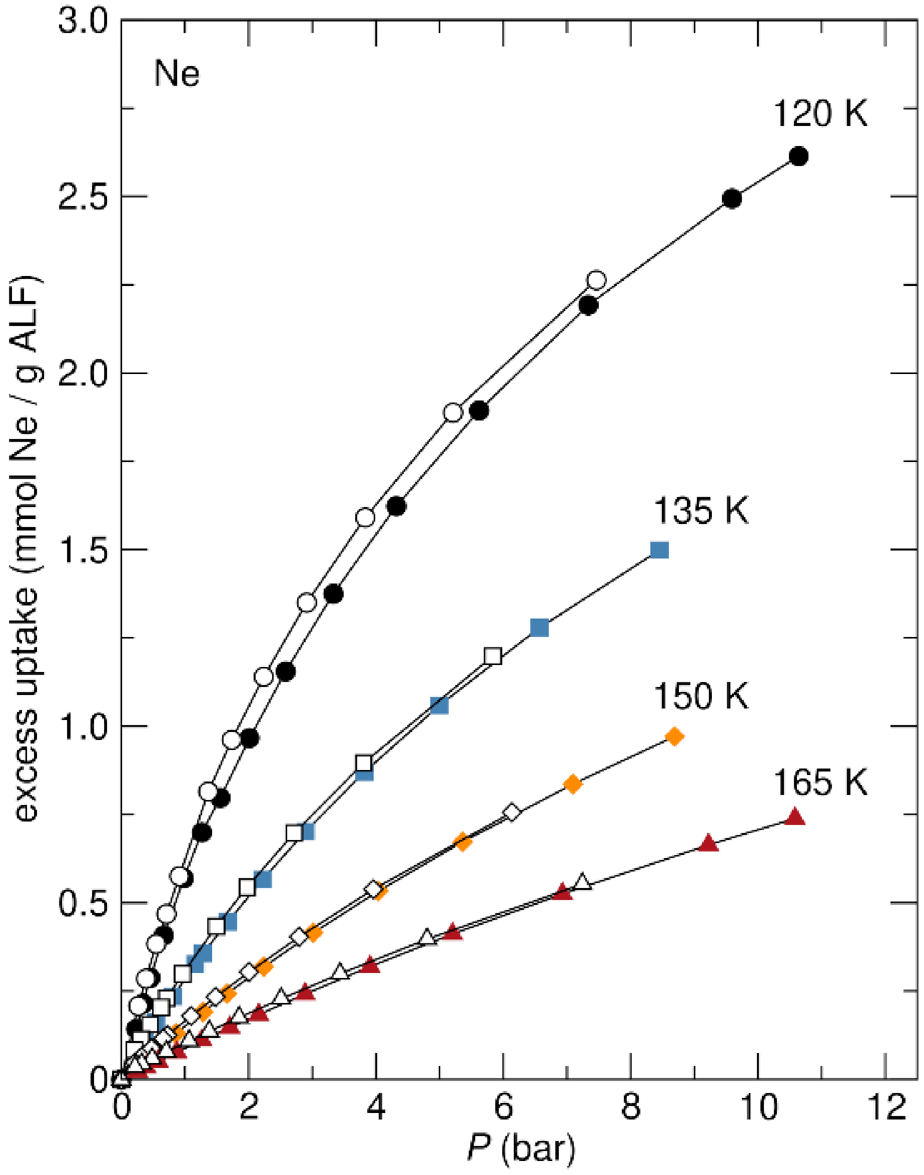

Figure S5: Ne excess isotherms between 120 K and 165 K. Closed symbols denote adsorption isotherms, open symbols denote desorption isotherms for each given temperature.

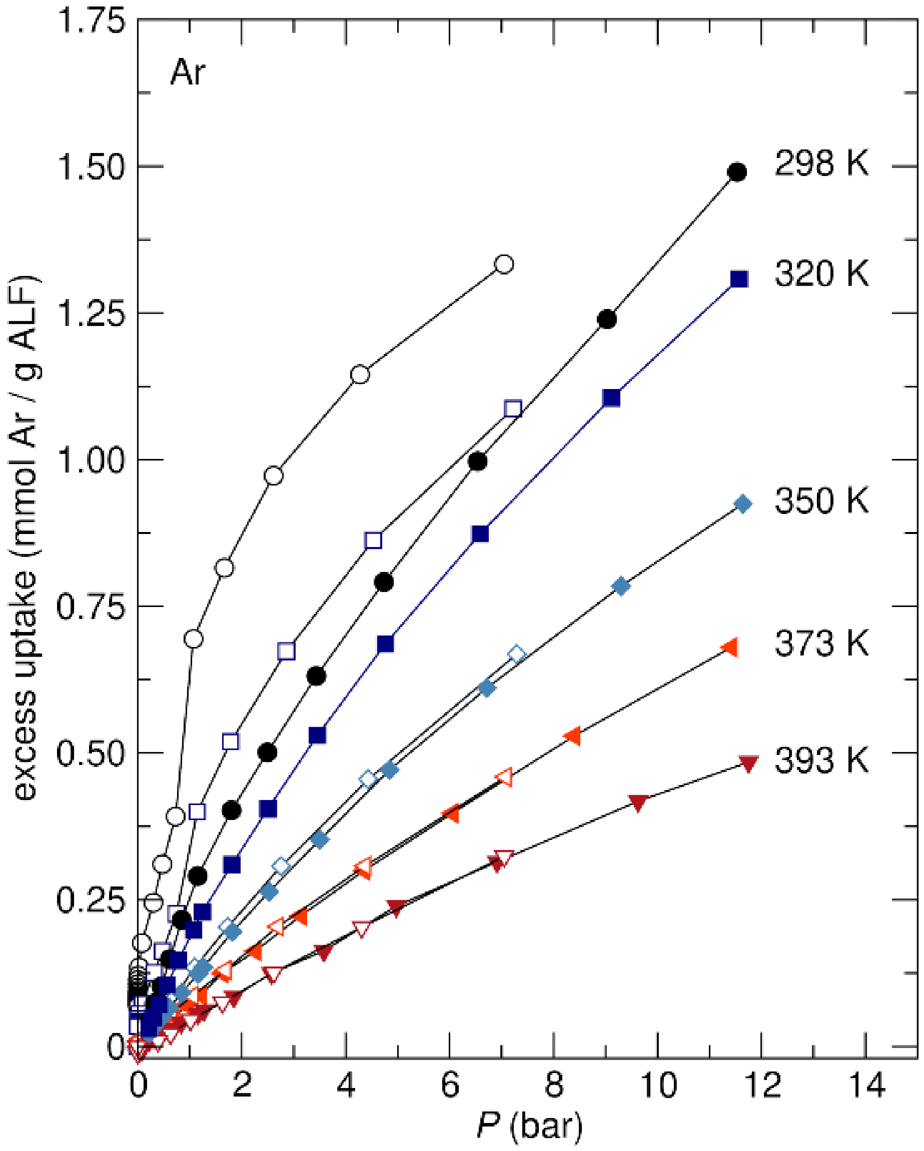

Figure S6: Ar excess isotherms between 298 K and 393 K. Closed symbols denote adsorption isotherms, open symbols denote desorption isotherms for each given temperature.

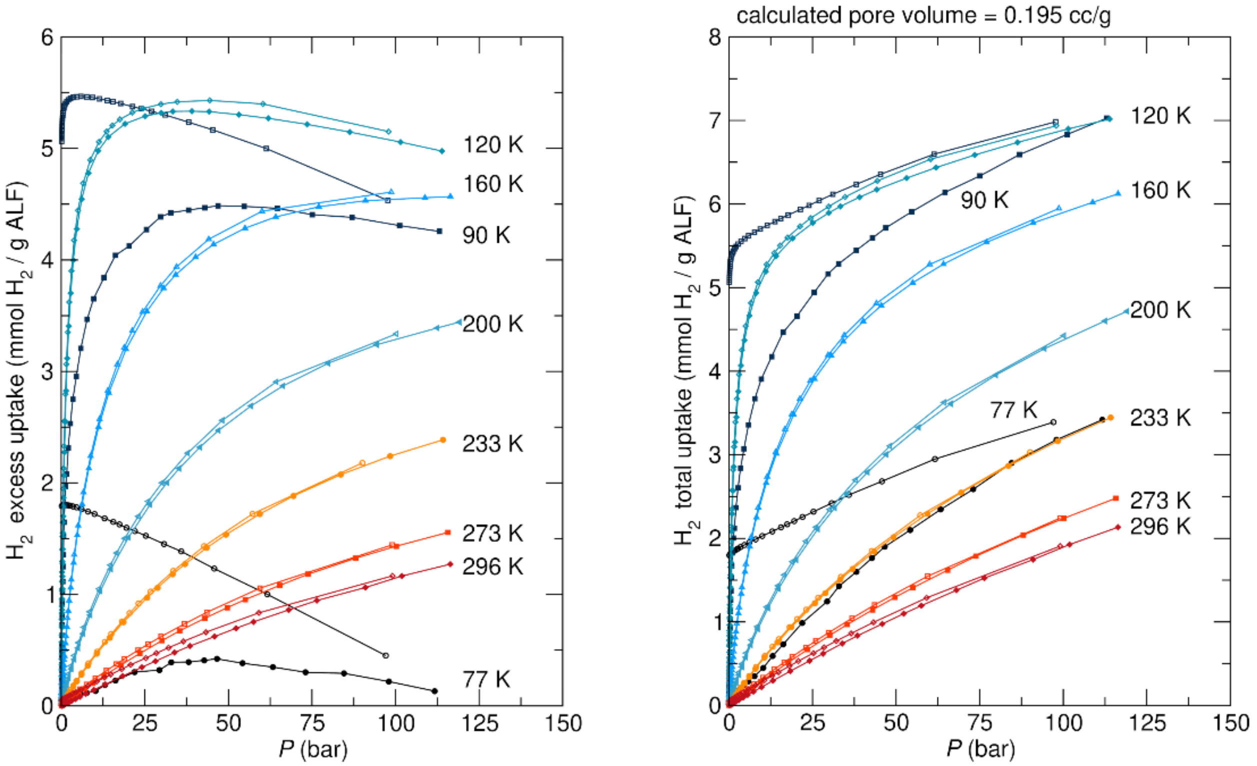

Figure S7: H_2 excess and total isotherms between 77 K and 296 K. Closed symbols denote adsorption isotherms, open symbols denote desorption isotherms for each given temperature.[3]

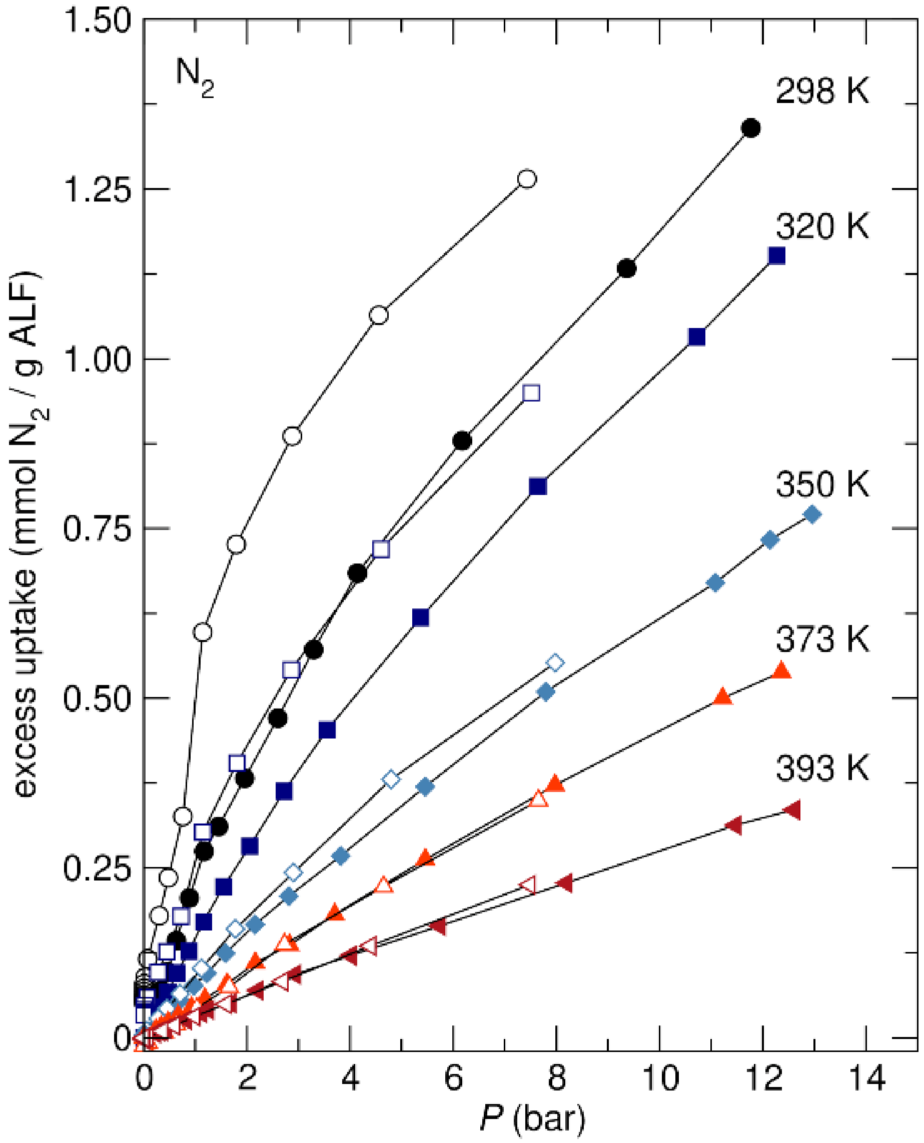

Figure S8: N_2 excess isotherms between 298 K and 393 K. Closed symbols denote adsorption isotherms, open symbols denote desorption isotherms for each given temperature.

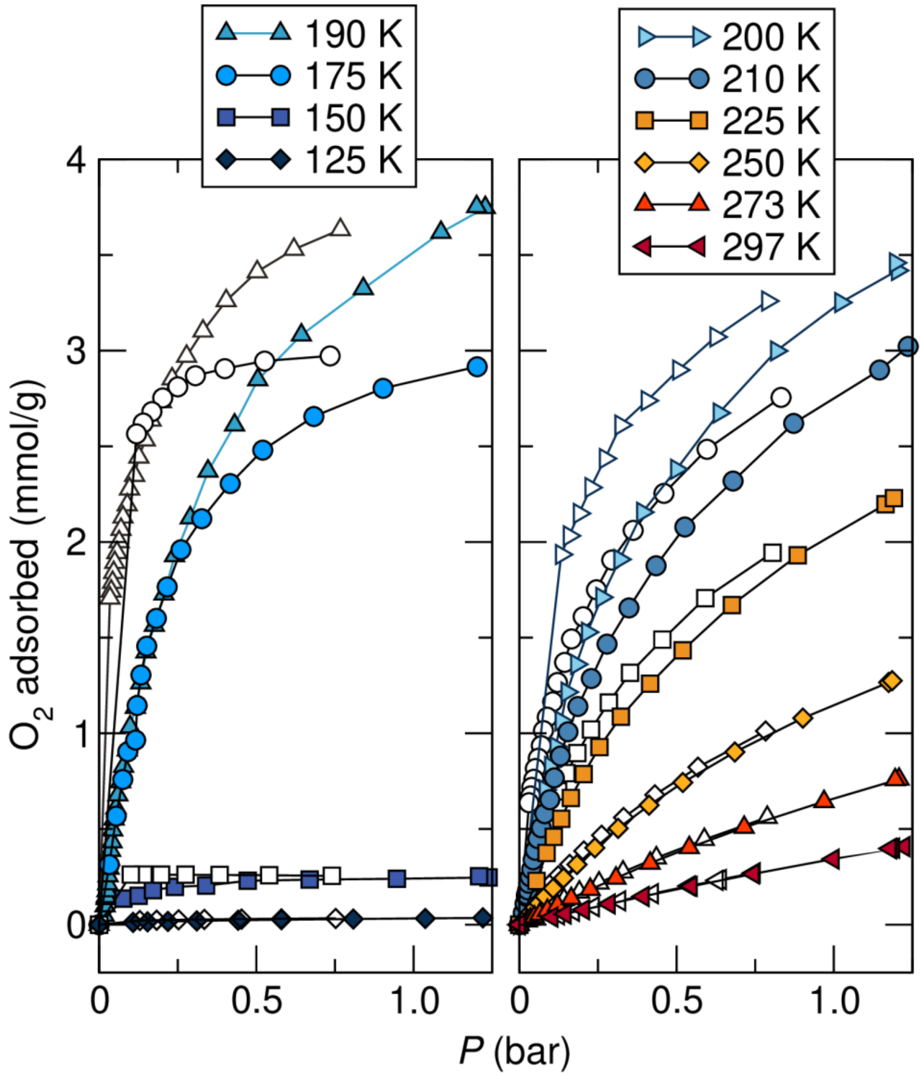

Figure S9: O_2 isotherms between 125 K and 297 K. Closed symbols denote adsorption isotherms, open symbols denote desorption isotherms for each given temperature.[2]

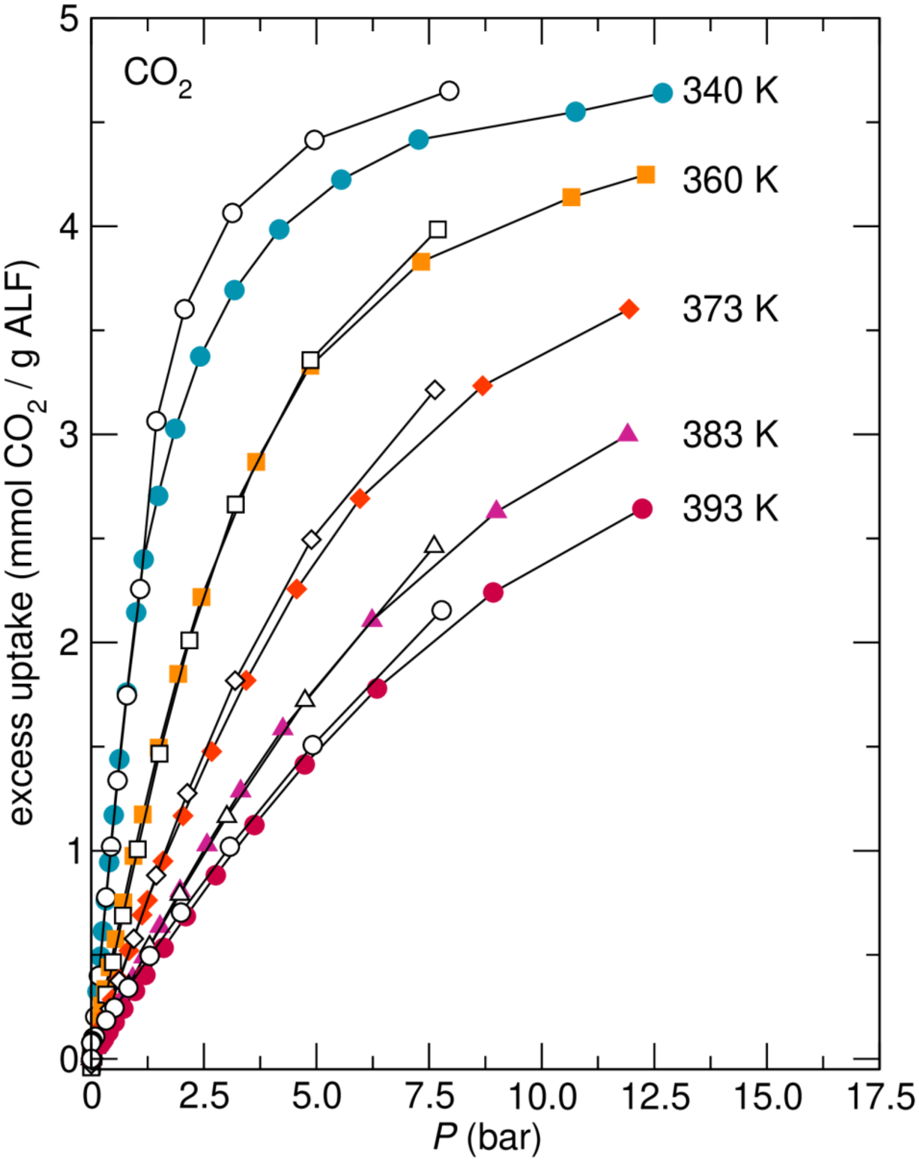

Figure S10: CO_2 excess isotherms between 340 K and 393 K. Closed symbols denote adsorption isotherms, open symbols denote desorption isotherms for each given temperature.

Kinetic adsorption profiles and fitting

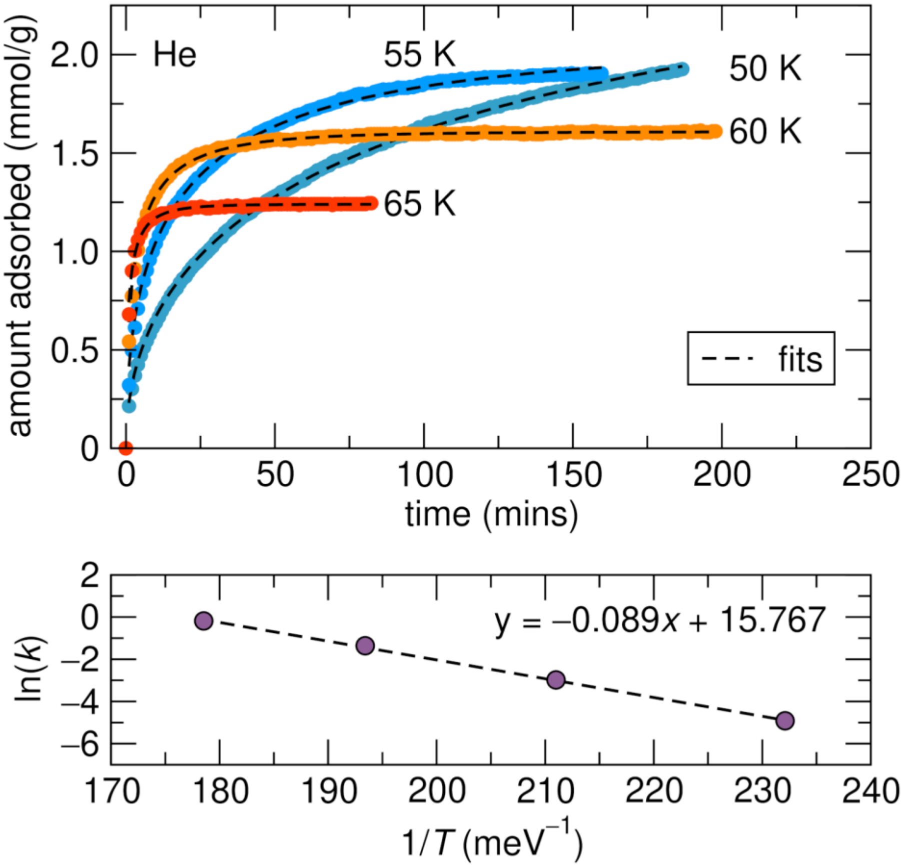

Figure S11: Top panel: Kinetic adsorption studies of He with ≈ 8 bar at various temperature alongside Avrami fits (results in Table S1) beneath lowest temperature He KIT. Bottom panel: Arrhenius fitting to obtain activation energy. E_a = 89 meV. Temperature values from measurements were converted to meV prior to fitting (1 meV = 11.6 K).

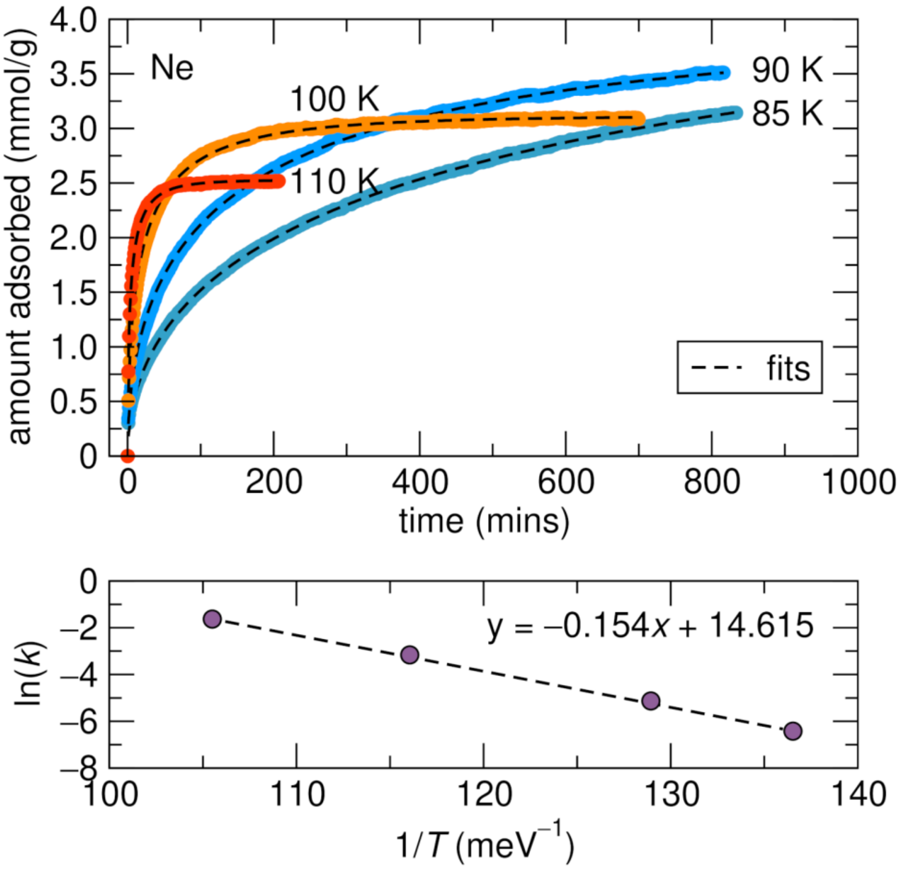

Figure S12: Top panel: Kinetic adsorption studies of Ne with ≈ 8 bar at various temperature alongside Avrami fits (results in Table S1) beneath lowest temperature Ne KIT. Bottom panel: Arrhenius fitting to obtain activation energy. E_a = 154 meV. Temperature values from measurements were converted to meV prior to fitting (1 meV = 11.6 K).

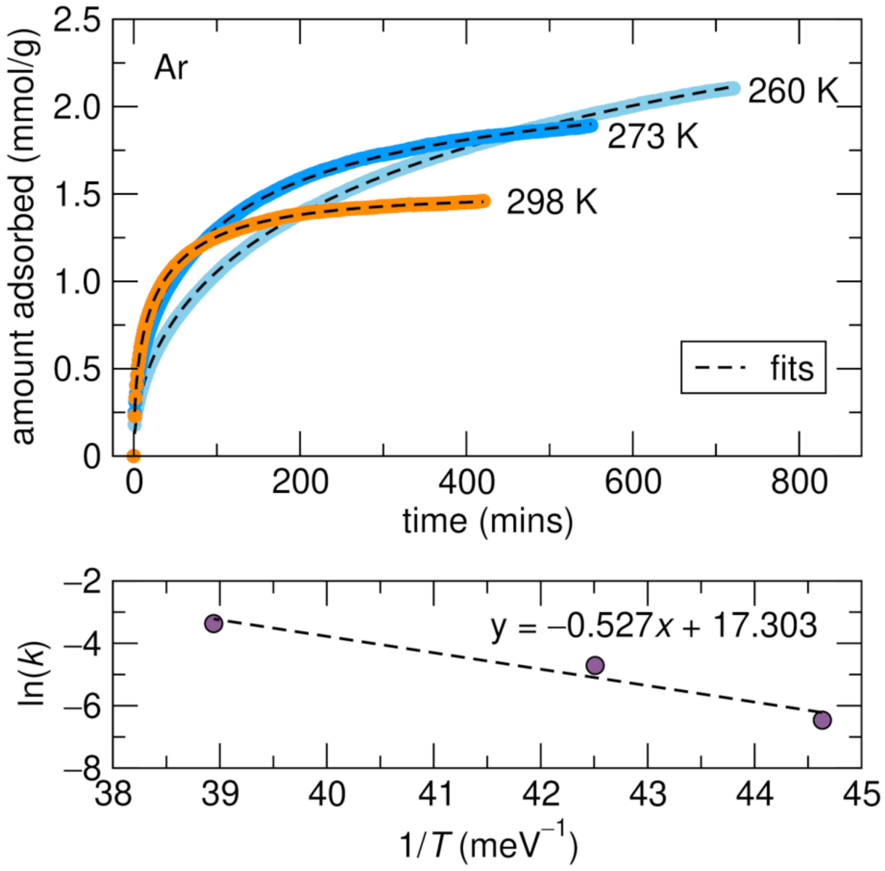

Figure S13: Top panel: Kinetic adsorption studies of Ar with ≈ 8 bar at various temperature alongside Avrami fits (results in Table S1) beneath lowest temperature Ar KIT. Bottom panel: Arrhenius fitting to obtain activation energy. E_a = 527 meV. Temperature values from measurements were converted to meV prior to fitting (1 meV = 11.6 K).

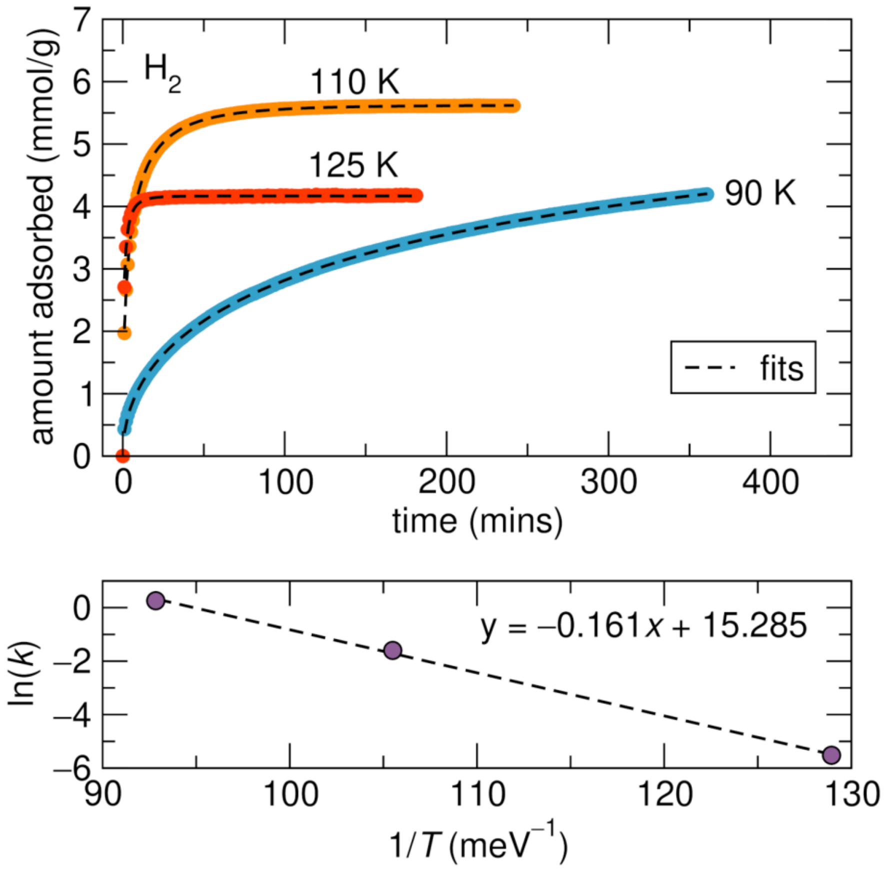

Figure S14: Top panel: Kinetic adsorption studies of H_2 with ≈ 8 bar at various temperature alongside Avrami fits (results in Table S1) beneath lowest temperature H_2 KIT. Bottom panel: Arrhenius fitting to obtain activation energy. E_a = 161 meV. Temperature values from measurements were converted to meV prior to fitting (1 meV = 11.6 K).

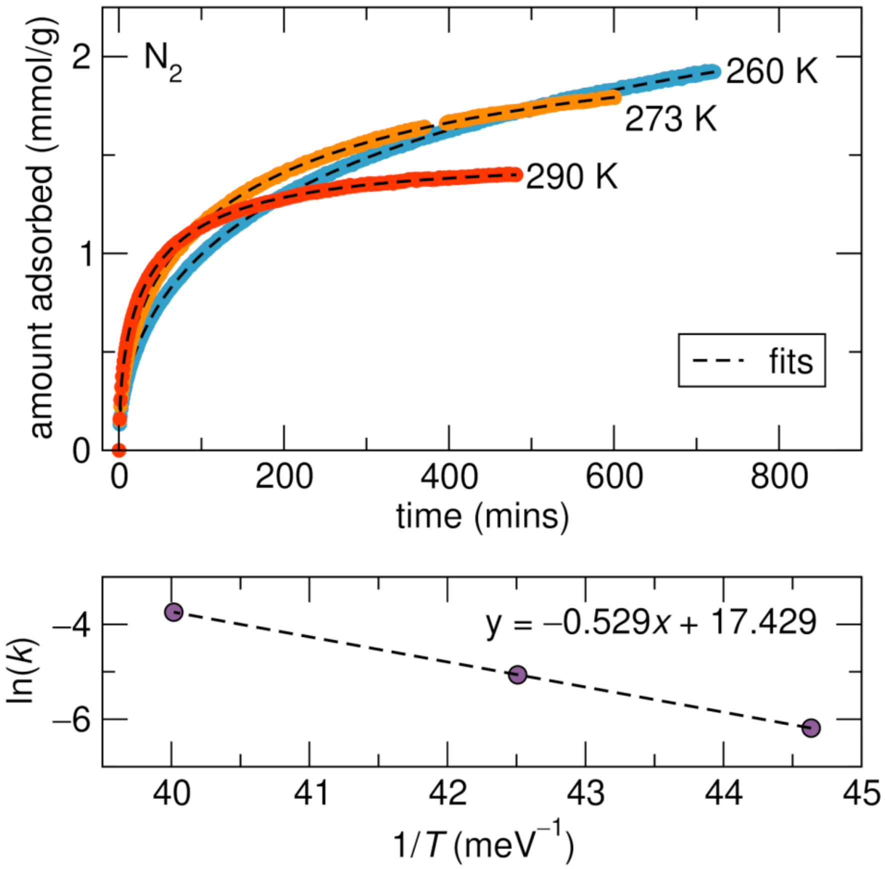

Figure S15: Top panel: Kinetic adsorption studies of N_2 with ≈ 8 bar at various temperature alongside Avrami fits (results in Table S1) beneath lowest temperature N_2 KIT. Bottom panel: Arrhenius fitting to obtain activation energy. E_a = 529 meV. Temperature values from measurements were converted to meV prior to fitting (1 meV = 11.6 K).

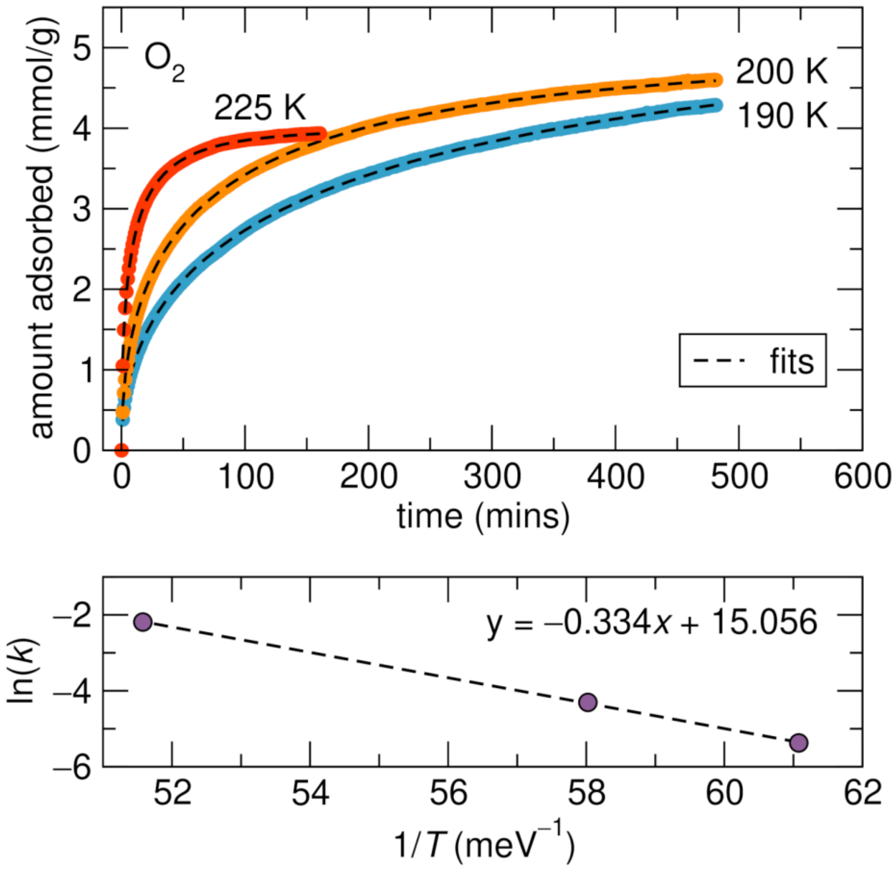

Figure S16: Top panel: Kinetic adsorption studies of O_2 with ≈ 8 bar at various temperature alongside Avrami fits (results in Table S1) beneath lowest temperature O_2 KIT. Bottom panel: Arrhenius fitting to obtain activation energy. E_a = 334 meV. Temperature values from measurements were converted to meV prior to fitting (1 meV = 11.6 K).

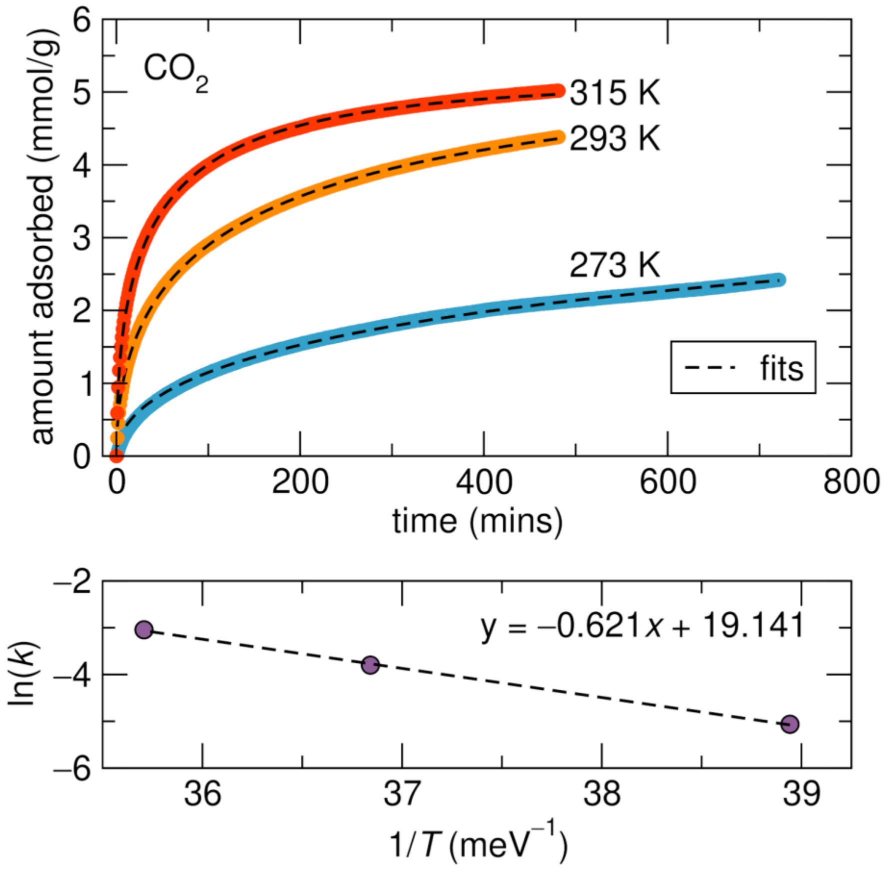

Figure S17: Top panel: Kinetic adsorption studies of CO_2 with ≈ 8 bar at various temperature alongside Avrami fits (results in Table S1) beneath lowest temperature CO_2 KIT. Bottom panel: Arrhenius fitting to obtain activation energy. E_a = 621 meV. Temperature values from measurements were converted to meV prior to fitting (1 meV = 11.6 K).

Gas	*Temp (K)*	*k*	q_m	E_a (meV)
He	50	0.00731	2.8166	89
	55	0.05095	2.0527	
	60	0.25664	1.6084	
	65	0.83261	1.2397	
Ne	85	0.00163	4.5733	154
	90	0.00592	3.9486	
	100	0.04235	3.1147	
	110	0.19479	2.5280	
Ar	260	0.00155	2.9536	527
	273	0.00900	2.1305	
	298	0.03455	1.4885	
H_2	90	0.00399	6.0104	161
	110	0.20144	5.6233	
	125	1.29850	4.1676	
O_2	190	0.00464	5.5320	334
	200	0.01348	4.9800	
	225	0.11212	3.9890	
N_2	260	0.00205	2.7296	529
	273	0.00632	2.0914	
	290	0.02366	1.4494	
CO_2	273	0.00105	4.1485	621
	298	0.00630	5.2864	
	315	0.02241	5.1635	

Table S1: Avrami fitting results for kinetic adsorption studies for gases with ALF. Activation energy (E_a) results obtained from Arrhenius fitting of Avrami fitting are also included.

Gas	*KIT 1 (K)*	*KIT 2 (K)*	*KIT 3 (K)*
He	66.8	--	--
Ne	114.7	147.4	--
Ar	309.8	344.3	371.6
H_2	125.1	154.2	--
O_2	216.7	251.4	--
N_2	310.2	351.8	--
CO_2	334.0	358.4	375.2
C_2H_2	291.0	383.8	--

Table S2: Experimental KITs resolved from TPD fitting (Figure 2, main text). Values have been rounded to first decimal place.

Diffraction

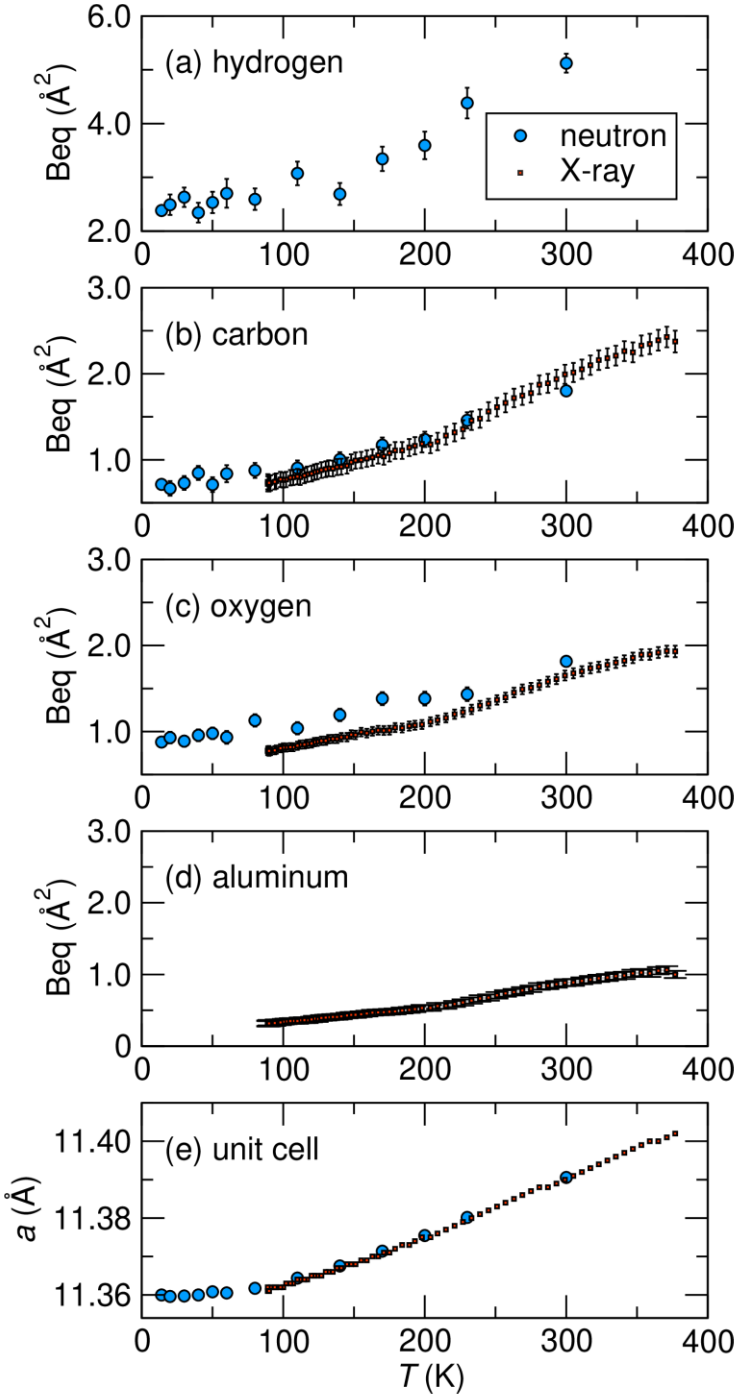

Figure S18: Select Rietveld refinement results of neutron (BT-1, NIST) and synchrotron X-ray (APS, 17-BM) data of bare ALF between 10 K and 380 K. X-ray hydrogen, and neutron aluminum Beq values are omitted as these values were fixed during refinements given the small scattering contributions of either atom for that respective method. Error bars shown denote 1 sigma. For symbols where the errors bars cannot be seen, the symbols are larger than obtained error bars.

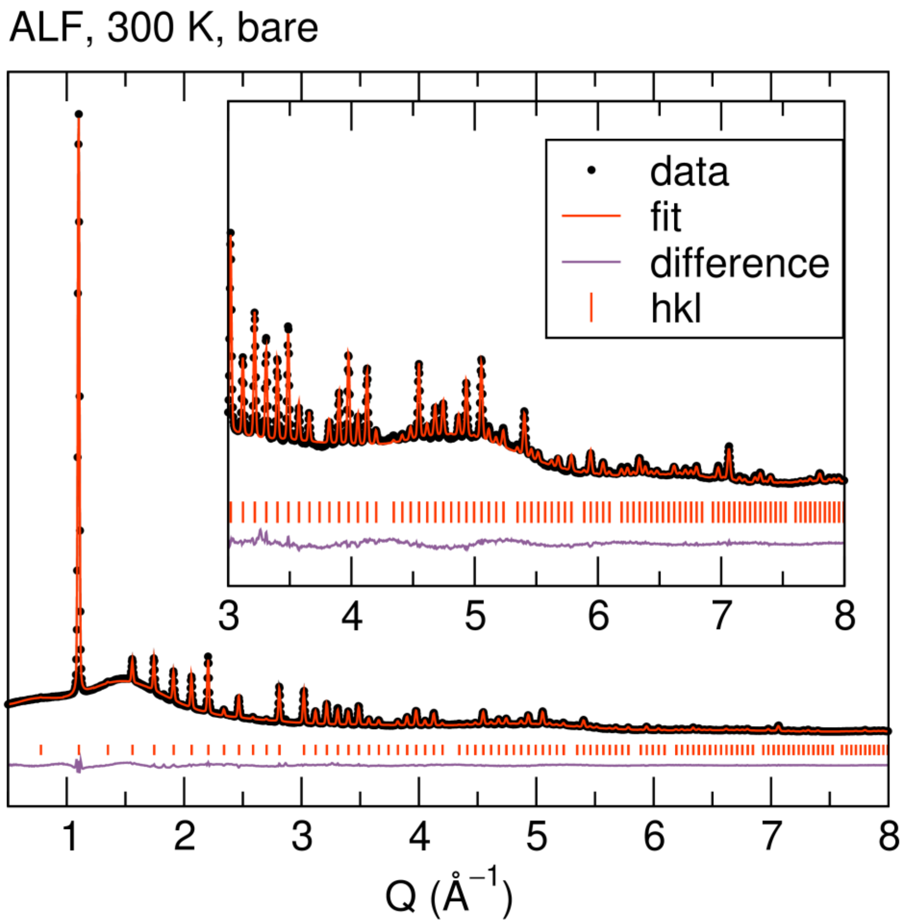

Figure S19: A representative sequential Rietveld refinement result from results shown in Figure S18 of synchrotron X-ray (17-BM, APS, λ = 0.24101 Å) data taken of bare ALF at 300 K. R_{wp} = 2.44 %.

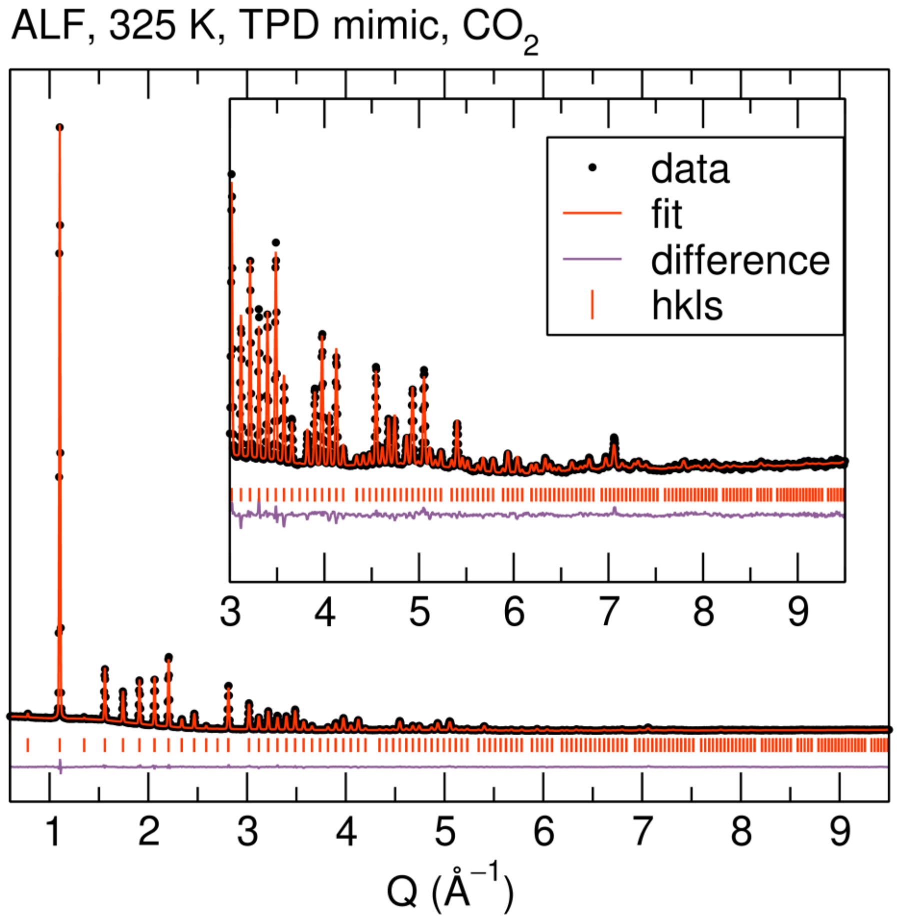

Figure S20: A representative sequential Rietveld refinement result from results shown in Figure S21. Data is of synchrotron X-ray (XPD, XLS-II, λ = 0.1821 Å) data taken of ALF loaded with CO_2 and heated, replicating TPD experiments. T = 325 K; R_{wp} = 2.24 %.

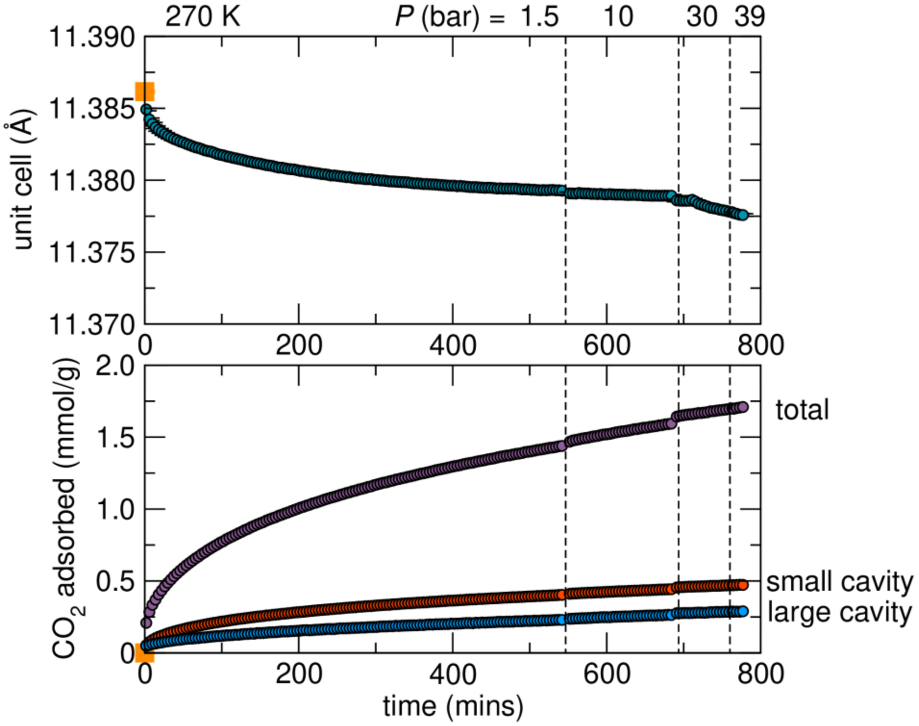

Figure S21: In-situ rapid acquisition X-ray diffraction with ALF at 270 K during CO_2 adsorption up to 39 bar (APS, 17-BM, λ = 0.24101 Å, ≈ 12.5 hours). The dashed lines denote where pressure was increased during monitoring. Orange square denotes activated ALF values. The tracking shows that even with pressure, when beneath the lowest KIT for CO_2 of 330 K, diffusion is greatly hindered. Small cavity (SC) and large cavity (LC) refer to the occupancies per cavity, and given there are three times as many SCs as LCs, the total population is equal to (3 × SC) + LC. The procedure for the diffraction experiment is described in the experimental section. Error values (1 sigma) for data points are smaller than the symbols used.

Thermal Expansion

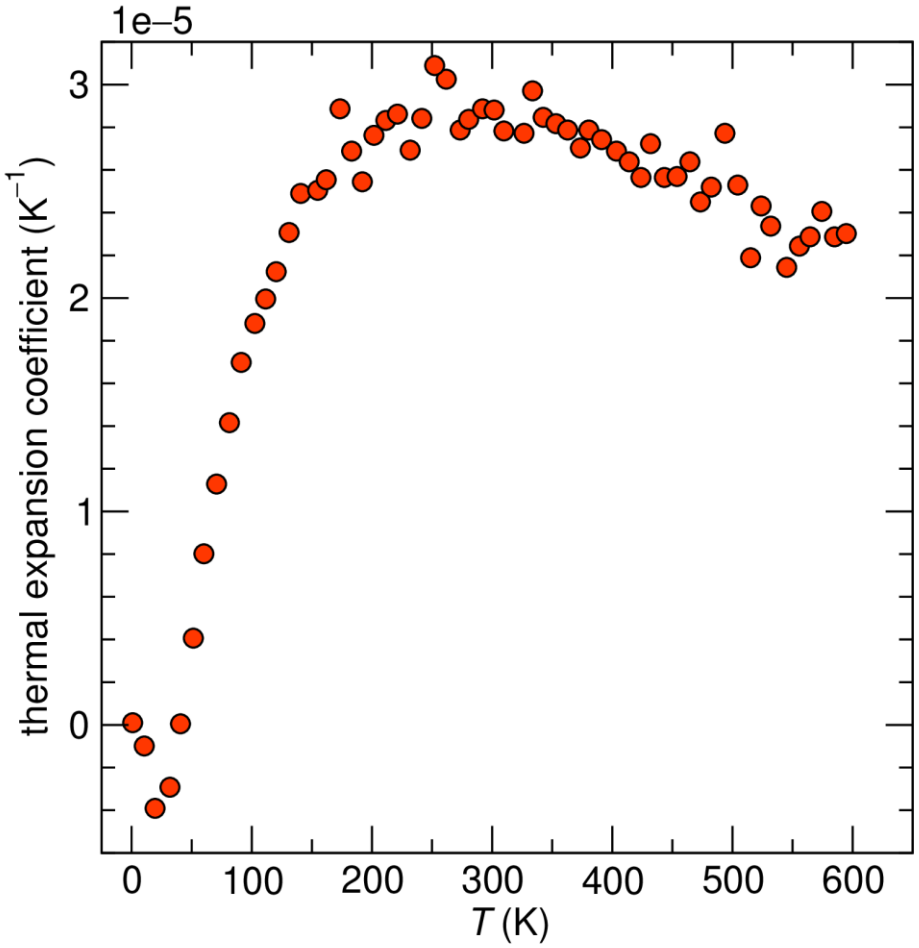

Figure S22: Calculated thermal expansion coefficients for ALF.[1]

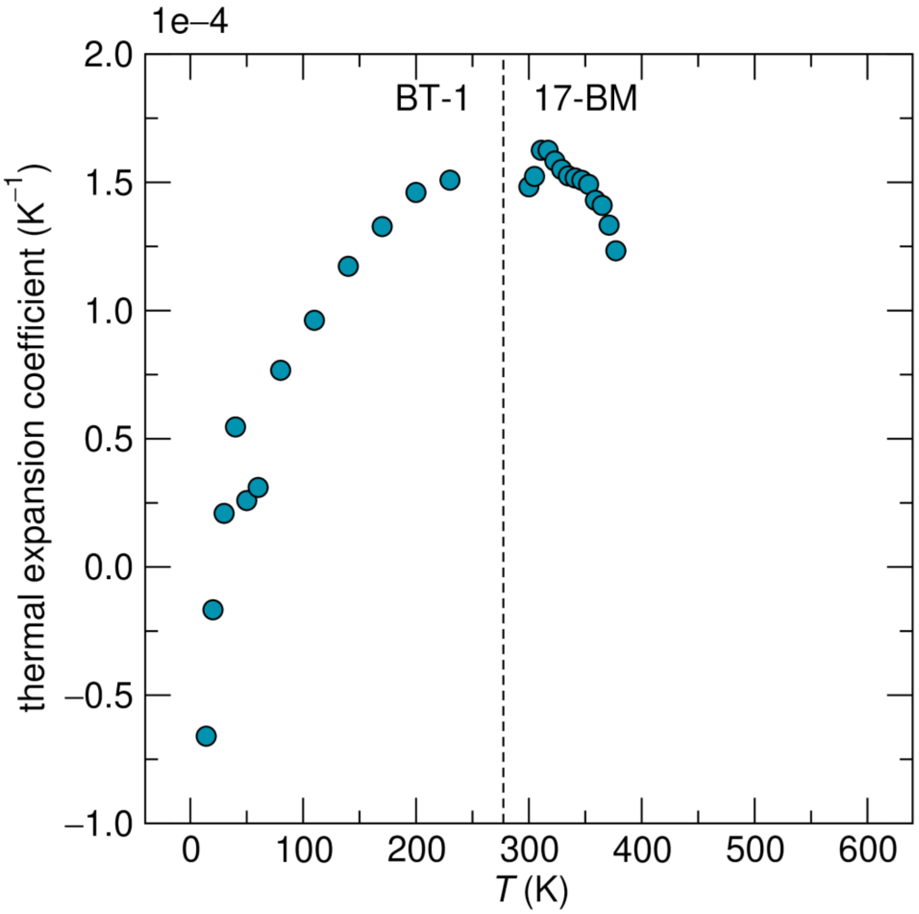

Figure S23: Experimental thermal expansion coefficients obtained from neutron diffraction data (NIST, BT-1) between 15 K and 240 K, and synchrotron X-ray diffraction data (APS, 17-BM) between 300 K and 380 K.

Raman Fitting

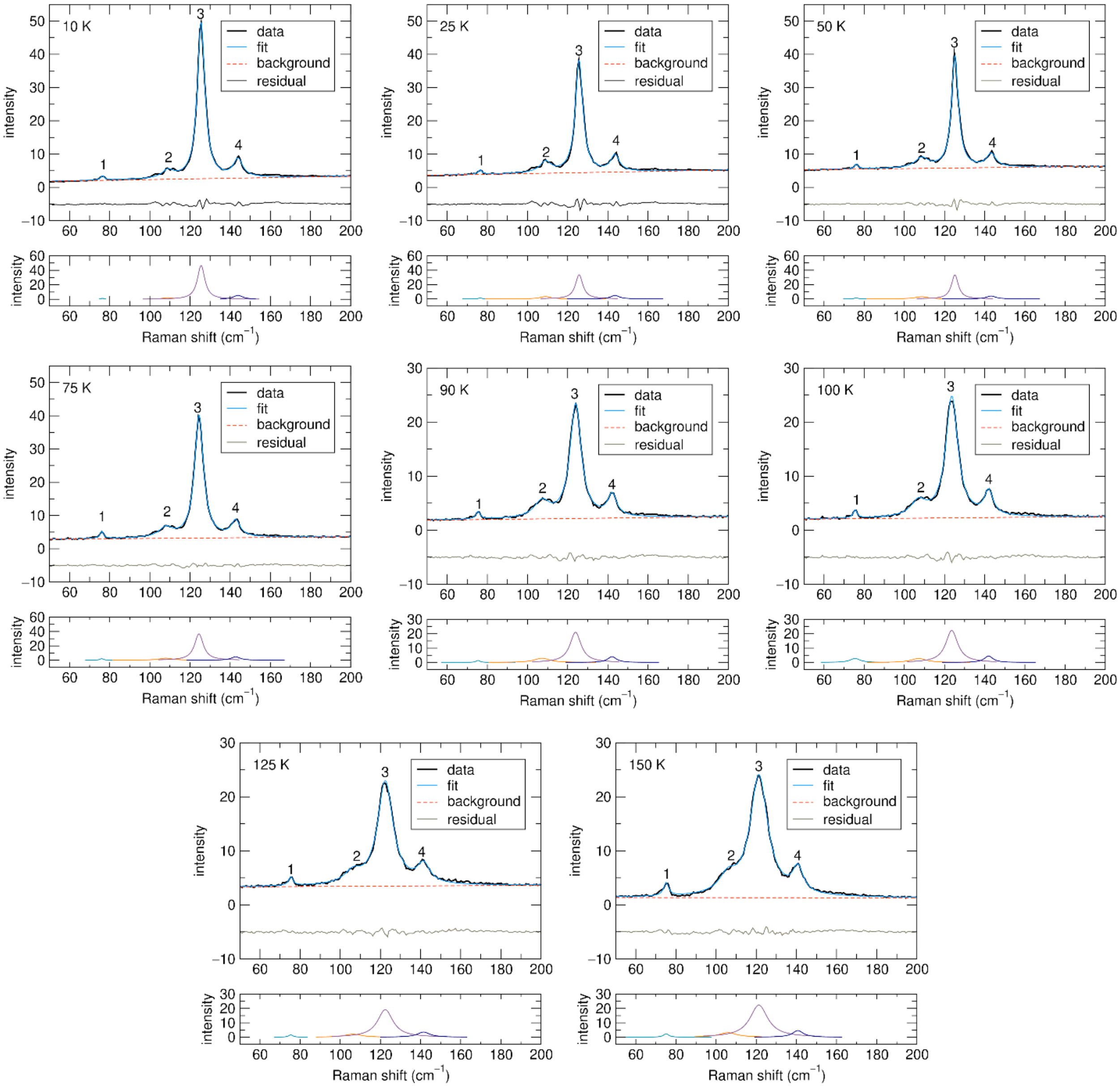

Figure S24: Raman fitting performed on peak areas **1-4** between 60 cm^{-1} and 200 cm^{-1} (Figure 3, main text). The bottom panel for each temperature subfigure illustrates the locations and intensities of the composite peaks. Each composite peak is a Lorentzian distribution.

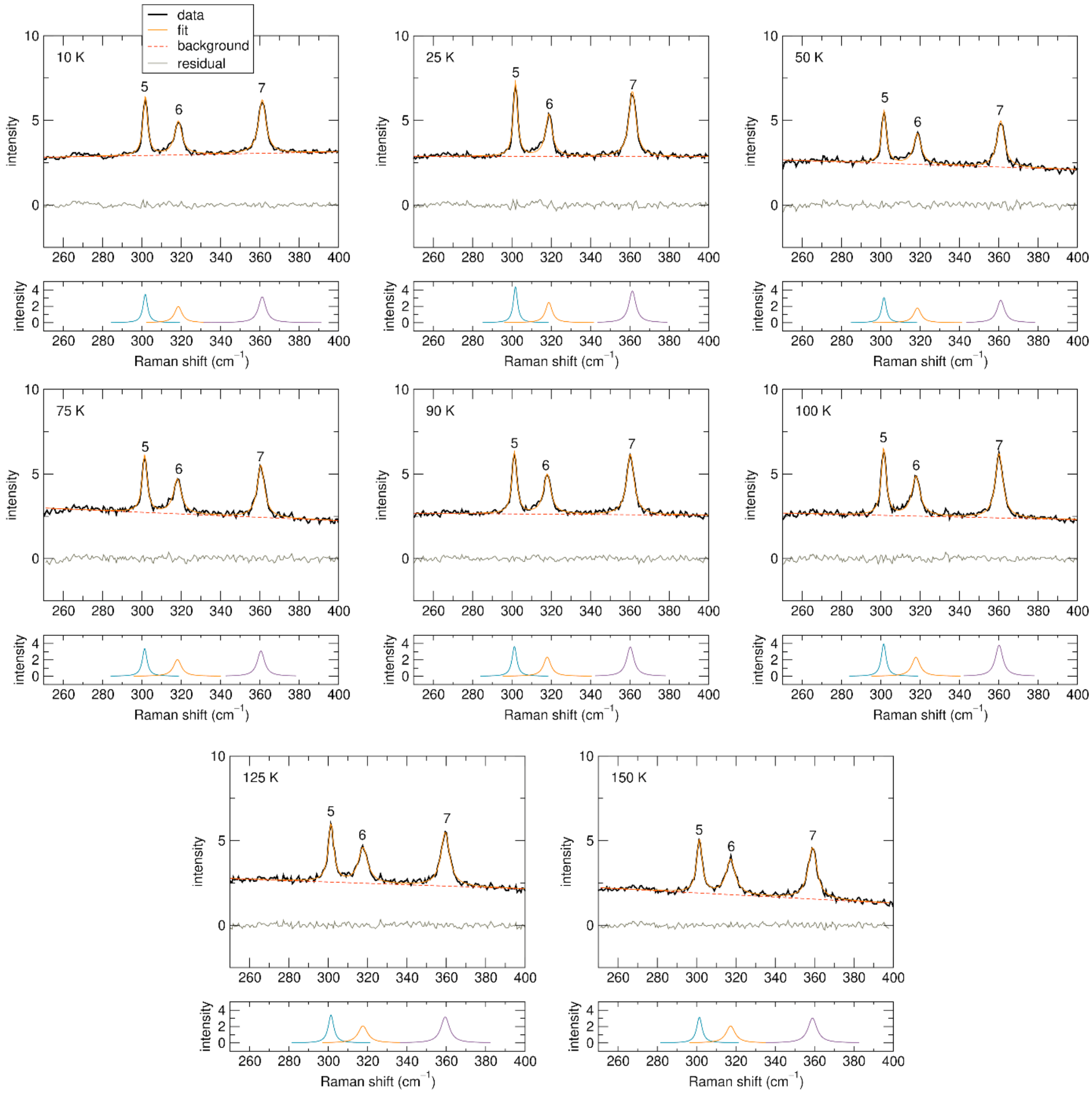

Figure S25: Raman fitting performed on peak areas **5-7** between 260 cm^{-1} and 400 cm^{-1} (Figure 3, see main text). The bottom panel for each temperature subfigure illustrates the locations and intensities of the composite peaks. Each composite peak is a Lorentzian distribution.

Neutron Spectroscopy

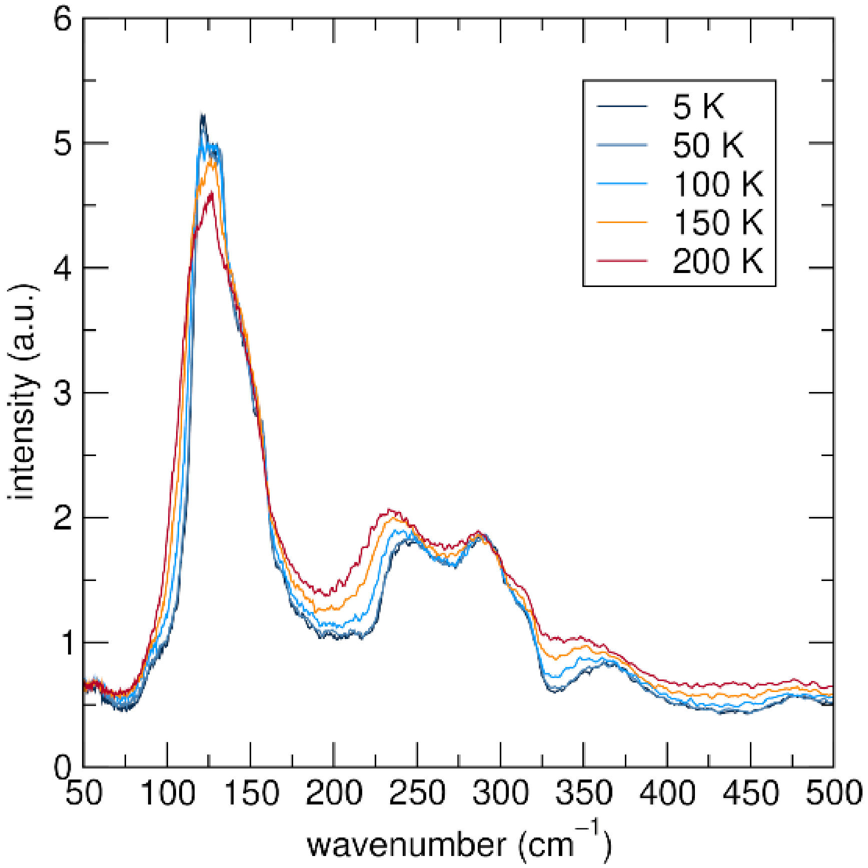

Figure S26: Inelastic neutron scattering spectra of bare ALF between 5 K and 200 K.

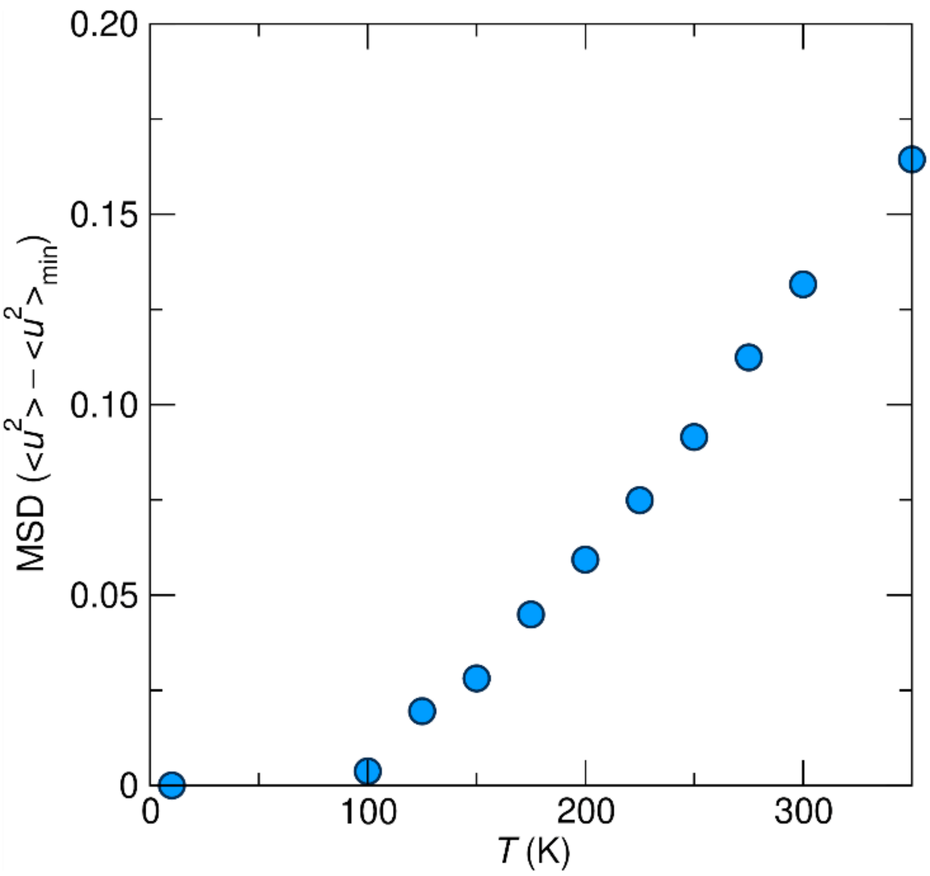

Figure S27: Quasielastic Neutron Scattering results on bare ALF graphed as a function of mean squared displacement versus temperature. Given QENS is incredibly sensitive to hydrogen atoms, this plot shows that at 100 K, the dynamics of the formate linkers grow exceedingly quickly as temperature is increased. This is supported by the trend noted from diffraction, INS, and Raman spectroscopy.

Density Functional Theory

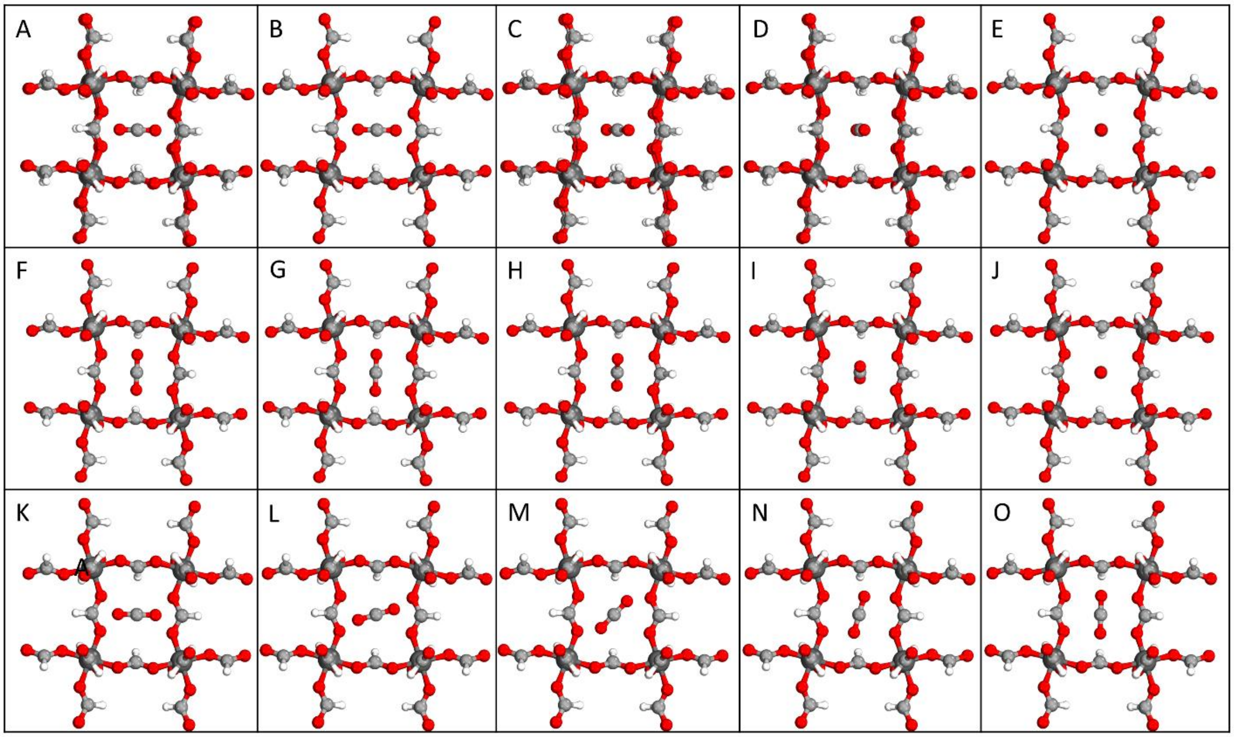

Figure S28: Atomistic view of the intrapore rotation of CO_2 in the SC of ALF. A-E: SC_Y-to-SC_X. F-J: SC_Z-to-SC_X. K-O: SC_Y-to-SC_Z

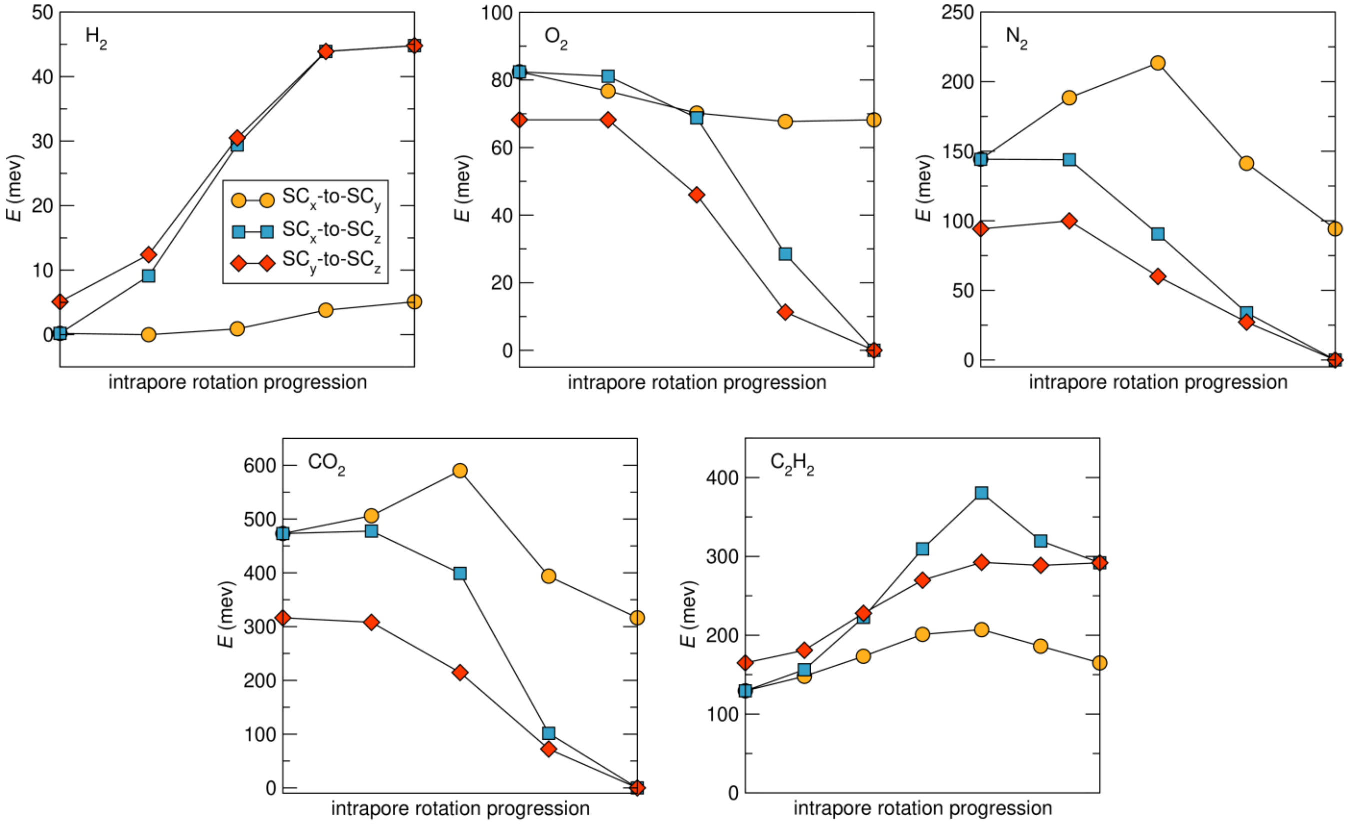

Figure S29: Energy diagrams for the intrapore rotation of H_2, O_2, N_2, CO_2, and C_2H_2 in the SC of ALF.

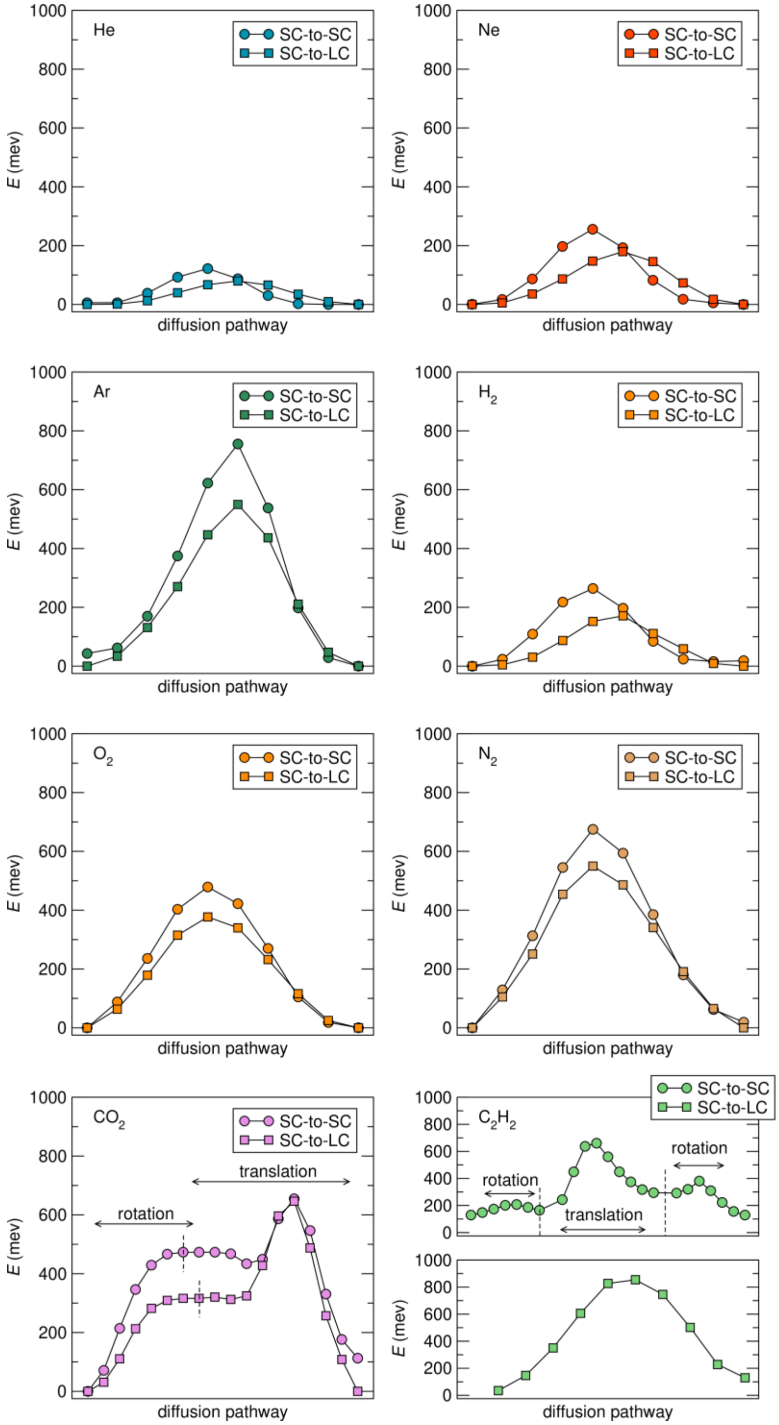

Figure S30: Energies of gas migration from SC-to-SC (circles) and SC-to-LC (squares) for the 8 different adsorbates. For the CO_2 graph, the dashed vertical lines denote the different points at which rotation of the molecule is complete. For the C_2H_2 SC-to-SC subplot, there are two periods of rotation as the molecule traverses between the SC's.

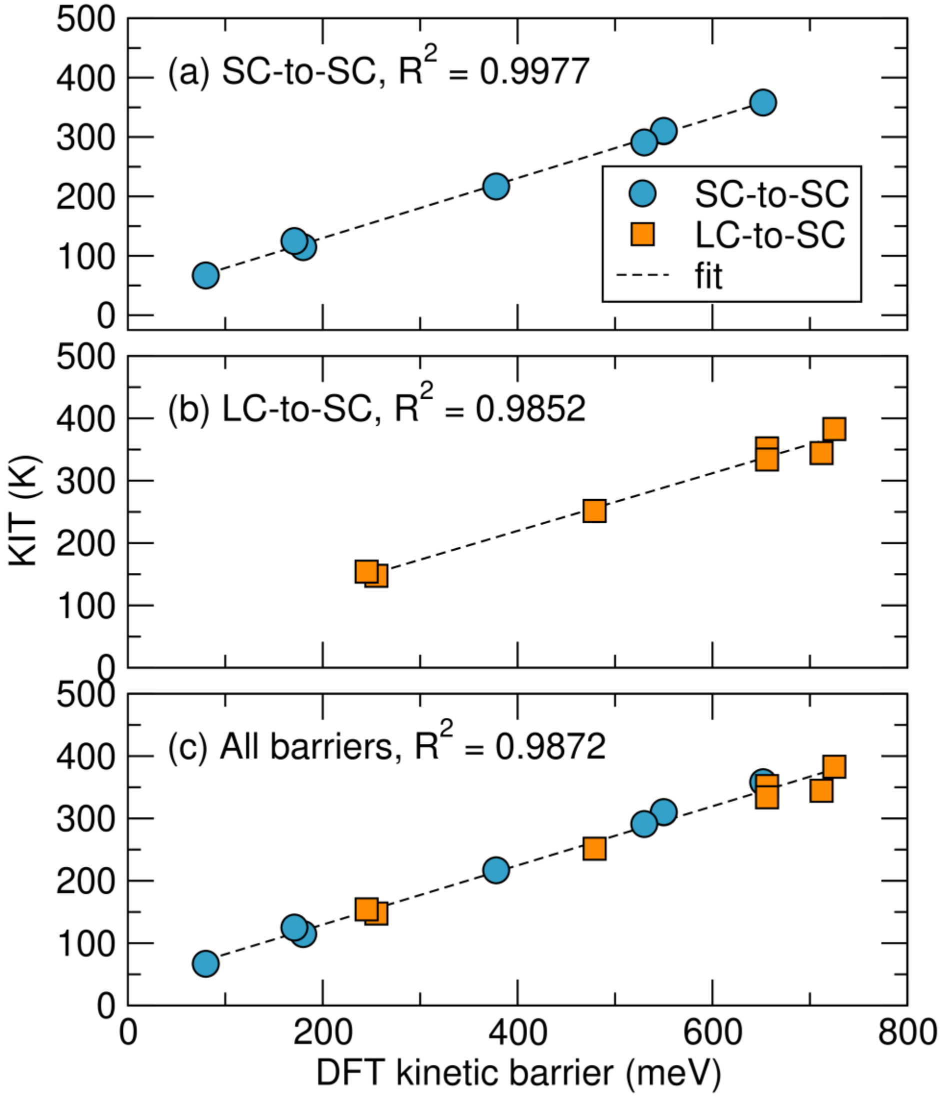

Figure S31: Graph illustrating the TPD derived KITs vs corresponding DFT SC-to-SC and LC-to-SC barriers. Fits (black dashed lines) are drawn illustrating the varying degrees of linearity between each, or all datasets. See. See Tables S2 and S3 for values used to assign SC-to-SC and LC-to-SC barriers to respective KITs. The KIT values plotted account for the first and second KITs found for ALF for each adsorbate.

Gas	*SC-to-SC*	*SC-to-LC*	*LC-to-SC*
He	80	122	116
Ne	180	256	255
Ar	550	756	712
H_2	171	264	245
O_2	378	479	479
N_2	550	675	656
CO_2	652	656	543
C_2H_2	530	854	725

Table S3: DFT calculated kinetic migration barriers between cavities (meV).

Gas	SC_x	SC_y	SC_z	*LC*
He	−49.2	−49.2	−49.2	−47.3
Ne	−98.2	−98.2	−98.2	−91.8
Ar	−196.9	−196.9	−196.9	−239.9
H_2	−134.8	−129.8	−90.1	−116.1
O_2	−151.9	−166.1	−234.2	−234.1
N_2	−141.3	−191.2	−285.5	−266.3
CO_2	−24.2	−132.6	−448.9	−336.2
C_2H_2	−316.718	−281.268	−154.328	−446.208

Table S4: DFT calculated binding energies (meV) for each adsorbate in the three SC orientations and LC.

Gas	*SC-to-SC*	*SC-to-LC*	*LC-to-SC*
He	7.72	11.77	11.19
Ne	17.37	24.70	24.60
Ar	53.07	72.94	68.70
H_2	16.50	25.47	23.64
O_2	36.47	46.22	46.22
N_2	53.07	65.13	63.29
CO_2	62.91	63.29	52.39
C_2H_2	51.14	82.40	69.95

Table S5: DFT calculated kinetic migration barriers between cavities (kJ/mol).

Gas	SC_x	SC_y	SC_z	*LC*
He	−4.75	−4.75	−4.75	−4.56
Ne	−9.47	−9.47	−9.47	−8.86
Ar	−19.00	−19.00	−19.00	−23.15
H_2	−13.01	−12.52	−8.69	−11.20
O_2	−14.66	−16.03	−22.60	−22.59
N_2	−13.63	−18.45	−27.55	−25.69
CO_2	−2.33	−12.79	−43.31	−32.44
C_2H_2	−30.56	−27.14	−14.89	−43.05

Table S6: DFT calculated binding energies (kJ/mol) for each adsorbate in the three SC orientations and LC.

Frequency (cm^{-1})	*Intensity (km mol^{-1})*	*Irreducible rep.*	*Mode type*
**(1)** 76.7280	0.42	E_g	Composed mode,<br>predominantly framework breathing
**(2)** 113.1244	0.40	F_g	Composed mode,<br>predominantly formate wagging
**(3)** 124.2644	70.49	E_g	Composed mode,<br>predominantly formate wagging
130.6857	12.94	F_g	Composed mode
139.8781	8.40	A_g	Composed mode
**(4)** 146.3309	1.89	F_g	Composed mode,<br>predominantly formate twisting
180.2138	0.01	A_g	Composed mode
208.0625	0.04	F_g	Composed mode
269.0706	1.77	E_g	Composed mode
284.7741	0.10	A_g	Composed mode
**(5)** 309.8459	16.36	F_g	Composed mode, predominantly Al-O_6<br>stretching, some formate rocking
**(6)** 315.8477	2.38	F_g	Composed mode, predominantly Al-O_6<br>scissoring, some formate rocking
328.2857	14.75	F_g	Composed mode, predominantly Al-O_6<br>scissoring, some formate rocking
**(7)** 366.7385	23.25	A_g	Composed mode, predominantly Al-O_6<br>scissoring, some formate rocking
448.8740	0.08	F_g	Al-O_6 symmetrical stretching
841.1657	5.39	F_g	O-C-O bending
845.7549	25.33	E_g	O-C-O bending
848.7237	14.07	A_g	O-C-O bending
1085.2359	0.62	F_g	H-C-O bending
1086.5447	0.04	A_g	H-C-O bending
1086.6094	17.07	E_g	H-C-O bending
1398.4222	259.83	F_g	H-C-O bending
1408.3253	2.25	F_g	H-C-O bending
1433.3592	0.08	F_g	C-O symmetrical stretching
1435.2761	310.56	E_g	C-O symmetrical stretching
1440.0194	776.00	A_g	C-O symmetrical stretching
1645.2993	1.15	F_g	C-O asymmetrical stretching
1765.4643	1.55	F_g	C-O asymmetrical stretching
3110.9348	1000.00	A_g	C-H symmetrical stretching
3112.4293	373.59	F_g	C-H symmetrical stretching
3114.1207	197.87	E_g	C-H asymmetrical stretching

Table S7: Simulated Raman modes and their intensities using dispersion-corrected hybrid (PBE0) density functional theory calculations of ALF in $Im\overline{3}$ (204). Irreducible representations of computed Raman modes are assigned. Bolded numbers in the frequency column refer to the modes tracked in Figure 3 of the main text.

References

(1) Evans, H. A.; Mullangi, D.; Deng, Z.; Wang, Y.; Peh, S. B.; Wei, F.; Wang, J.; Brown, C. M.; Zhao, D.; Canepa, P.; Cheetham, A. K. Aluminum Formate, $Al(HCOO)_3$: An Earth-Abundant, Scalable, and Highly Selective Material for CO_2 Capture. *Sci. Adv.* **2022**, *8* (44), eade1473. https://doi.org/10.1126/sciadv.ade1473.

(2) Mullangi, D.; Evans, H. A.; Yildirim, T.; Wang, Y.; Deng, Z.; Zhang, Z.; Mai, T. T.; Wei, F.; Wang, J.; Hight Walker, A. R.; Brown, C. M.; Zhao, D.; Canepa, P.; Cheetham, A. K. Noncryogenic Air Separation Using Aluminum Formate $Al(HCOO)_3$ (ALF). *J. Am. Chem. Soc.* **2023**, *145* (17), 9850–9856. https://doi.org/10.1021/jacs.3c02100.

(3) Evans, H. A.; Yildirim, T.; Peng, P.; Cheng, Y.; Deng, Z.; Zhang, Q.; Mullangi, D.; Zhao, D.; Canepa, P.; Breunig, H. M.; Cheetham, A. K.; Brown, C. M. Hydrogen Storage with Aluminum Formate, ALF: Experimental, Computational, and Technoeconomic Studies. *J. Am. Chem. Soc.* **2023**, *145* (40), 22150–22157. https://doi.org/10.1021/jacs.3c08037.

(4) Zhang, Z.; Deng, Z.; Evans, H. A.; Mullangi, D.; Kang, C.; Peh, S. B.; Wang, Y.; Brown, C. M.; Wang, J.; Canepa, P.; Cheetham, A. K.; Zhao, D. Exclusive Recognition of CO_2 from Hydrocarbons by Aluminum Formate with Hydrogen-Confined Pore Cavities. *J. Am. Chem. Soc.* **2023**, *145* (21), 11643–11649. https://doi.org/10.1021/jacs.3c01705.

(5) Shirzad, K.; Viney, C. A Critical Review on Applications of the Avrami Equation beyond Materials Science. *J. R. Soc. Interface* **2023**, *20* (203), 20230242. https://doi.org/10.1098/rsif.2023.0242.
